# METTL3-Driven m6A Epigenetic Remodeling of lncRNA-AU020206 Stabilizes SLC7A11 via YTHDC2 Attenuates Apoptosis and Ferroptosis in Cerebral Ischemia/Reperfusion Injury

**DOI:** 10.3390/biom15101353

**Published:** 2025-09-24

**Authors:** Hao Zhang, Yajin Guan, Meng Li, Yilin Wu, Xiaoou Sun

**Affiliations:** 1 Institute of Biomedical and Pharmaceutical Sciences, Guangdong University of Technology, Guangzhou 510006, China; lm135837@163.com (M.L.); 17docwuyilin@smu.edu.cn (Y.W.); 2 Guangzhou Huateng Bioscience Corporation, Guangzhou 510700, China; guanyajin@htscience.com

**Keywords:** METTL3, YTHDC2, SLC7A11, lncRNA-AU020206, ferroptosis, ischemia/reperfusion

## Abstract

Cerebral ischemia/reperfusion (I/R) injury is a devastating neurological disorder with limited treatment options. Emerging evidence suggests that the N6-methyladenosine (m6A) modification and its regulatory factors play pivotal roles in the pathophysiology of I/R. This study aimed to elucidate the function of METTL3-mediated m6A modification of the long non-coding RNA (lncRNA) AU020206 in ferroptosis during cerebral I/R injury and to identify potential molecular targets for neuroprotection. A murine model of middle cerebral artery occlusion/reperfusion (MCAO/R) and N2a cells subjected to oxygen–glucose deprivation/reoxygenation (OGD/R) were established to assess m6A levels and ferroptosis-related changes. Effects of METTL3 overexpression and lncRNA-AU020206 silencing on neuronal apoptosis, inflammation, and ferroptosis were investigated in vitro and in vivo. The interaction between lncRNA-AU020206 and YTHDC2 and the resulting regulation of SLC7A11 mRNA stability and GPX4 expression were evaluated using molecular and biochemical assays. Both MCAO/R mice and OGD/R-treated N2a cells exhibited decreased m6A levels and upregulation of lncRNA-AU020206 accompanied by enhanced ferroptosis. METTL3 overexpression increased the m6A modification of AU020206, promoting its degradation and attenuating neuronal injury, whereas silencing AU020206 or overexpressing YTHDC2 decreased SLC7A11 mRNA stability and enhanced ferroptosis. Restoring the expression of SLC7A11/GPX4 can enhance cell viability, alleviate neuronal apoptosis, and reduce Fe^2+^ overload. Disruption of the METTL3–AU020206–YTHDC2 axis abolished these neuroprotective effects. METTL3-mediated m6A modification of lncRNA-AU020206 restrained ferroptosis and neuronal injury in cerebral I/R by maintaining the stability of the SLC7A11/GPX4 axis via interactions with YTHDC2. Targeting this epitranscriptomic signalling pathway may represent a promising therapeutic strategy for the treatment of ischemic stroke and related neurological disorders.

## 1. Introduction

Ischemic stroke accounts for nearly 80% of all cerebrovascular events worldwide, and remains the primary cause of long-term disability and mortality [1]. In China, large-scale epidemiological studies report an incidence of approximately 1114 cases per 100,000 person-years and close to 1.1 million stroke-related deaths annually, figures that constitute almost one-third of the global toll [2,3]. Currently, vessel recanalization strategies, such as intravenous thrombolysis and mechanical thrombectomy, are the only therapies with confirmed clinical benefits; however, their effectiveness is circumscribed by an exceptionally narrow therapeutic window, excluding a substantial proportion of patients [4,5]. Compounding this limitation, the delayed restoration of perfusion can exacerbate tissue injury through cerebral ischemia/reperfusion injury (CIRI). CIRI is characterized by mitochondrial dysfunction and excessive reactive oxygen species (ROS) that drive lipid peroxidation, protein carbonylation, and DNA fragmentation, thereby activating multiple regulated cell death programmes, including apoptosis, necroptosis, and ferroptosis, all of which aggravate neurological outcomes [6]. Consequently, elucidating the molecular circuitry underlying CIRI are indispensable steps toward developing next-generation neuroprotective strategies that can be deployed alongside recanalization to improve functional recovery after stroke.

Epitranscriptomics, the exploration of chemical modifications of cellular RNA-has emerged as a crucial layer of gene regulatory control with broad relevance to human pathology [7]. To date, more than 170 distinct RNA modifications have been identified, of which N6-methyladenosine (m6A) is the most abundant in higher eukaryotes and is especially enriched in the mammalian central nervous system [8]. The deposition, removal, and interpretation of m6A markers constitute a tightly regulated, reversible process orchestrated by “writers” (e.g., the methyltransferase complex centred on METTL3, METTL14), “erasers” (demethylases, such as FTO and ALKBH5), and a suite of YTH-domain–containing “readers” that translate the chemical mark into functional outcomes [9,10]. Recently, stroke biology has entered the field of epitranscriptomics. Accumulating evidence indicates that m6A regulators modulate key post-ischaemic events. For example, METTL3 activity shapes axonal regrowth, neuronal survival, neurogenesis, and the neuroinflammatory response following cerebral ischemia [11].

Long non-coding RNAs (lncRNAs) regulate gene expression through diverse mechanisms and are increasingly recognized as contributors to brain injury [12]. m6A modifications can alter lncRNA stability and interactions with RNA-binding proteins [13]. Among m6A readers, YTHDC2 modulates RNA stability and translation, yet its role in CIRI remains poorly understood [14]. Although YTHDC2 is essential for general RNA metabolism, its contribution to neuronal viability and pathogenesis of ischaemic stroke remains poorly characterized [15]. In the present study, we indicate that increased m6A deposition is the principal driver of lncRNA-AU020206 accumulation in oxygen–glucose deprivation/reoxygenation (OGD/R) neuronal cultures and in a middle cerebral artery occlusion/reperfusion (MCAO/R) rat model. Bioinformatic analyses predicted m6A sites in AU020206 and its potential interaction with YTHDC2, raising the possibility that this lncRNA may influence neuronal fate through post-transcriptional regulation. METTL3, a core m6A “writer”, catalyzes this modification, enhancing AU020206 stability and enabling it to orchestrate downstream signalling via preferential recruitment of YTHDF2. Collectively, our findings strengthen the concept that a “METTL3–m6A–lncRNA–reader” cascade constitutes a critical layer of post-stroke regulation and highlight lncRNA-centred epitranscriptomic networks as promising therapeutic targets for cerebral ischemia–reperfusion injury.

Ferroptosis is an iron-dependent, non-apoptotic mode of regulated cell death, characterized by glutathione (GSH) depletion, inactivation of glutathione peroxidase 4 (GPX4), and unchecked accumulation of phospholipid hydroperoxides within cellular membranes [16]. This process is initiated when intracellular labile iron catalyzes Fenton-type reactions that generate ROS, thereby driving the peroxidation of polyunsaturated fatty acid (PUFA)-containing phospholipids and disrupting redox homeostasis [17]. Central to ferroptosis control is the cystine/glutamate antiporter System Xc^−^, whose light chain, SLC7A11, imports extracellular cystine for GSH synthesis; repression or degradation of SLC7A11 curtails GSH availability and renders cells extremely sensitive to lipid peroxidation [18]. Epitranscriptomic m6A marks can fine-tune ferroptotic susceptibility by modulating the stability and translation of ferroptosis-related transcripts [19]. In particular, YTHDC2, a cytoplasmic m6A “reader” endowed with RNA helicase activity, facilitates decay or translational remodelling of its targets in an m6A-dependent fashion, yet its contribution to ferroptosis, especially in the context of cerebral I/R injury, is unexplored [20]. Bioinformatic interrogation of our transcriptome datasets uncovered a potential YTHDC2 interaction site within the 3′-UTR of SLC7A11, implying that YTHDC2 may regulate the antioxidant barrier in neurons by directly engaging SLC7A11 mRNA.

In the present study, we aimed to decipher the biological function and mechanistic scaffold of lncRNA AU020206 in ferroptosis-driven neuronal injury following cerebral I/R. Using OGD/R in N2a cells and a murine MCAO/R model, we observed a pronounced elevation in METTL3 expression accompanied by a surge in m6A-modified AU020206. MeRIP-qPCR and actinomycin-D decay assays confirmed that METTL3-catalyzed m6A deposition substantially extended the half-life of AU020206. RNA-immunoprecipitation and dual-luciferase reporter analyses further demonstrated that AU020206 functions as a molecular scaffold, recruiting the YTHDC2 to the 3′-UTR of SLC7A11 mRNA, thereby shielding SLC7A11 transcripts from degradation and preserving SLC7A11 activity. Functionally, the overexpression of either METTL3 or AU020206 attenuated lipid-ROS accumulation, GPX4 depletion, and neuronal death, whereas knockdown of YTHDC2 or SLC7A11 removed these protective effects. Collectively, our findings identified a METTL3/AU020206/YTHDC2/SLC7A11 axis that curbs ferroptotic injury after cerebral I/R and highlight epitranscriptomic modulation of lncRNA stability as a promising therapeutic avenue for ischaemic stroke.

## 2. Methods

### 2.1. Animals and Establishment of Ischemic Stroke Model

All procedures were performed in accordance with the Guidelines for the Care and Use of Laboratory Animals and were approved by the Animal Ethics Committee of Guangzhou Huateng Biomedical Technology Co., Ltd. (approval no. HTSW221143, 19 April 2023). Twenty-four male C57BL/6J mice (8–10 w old, 22–26 g) were obtained from the same institution’s specific pathogen-free (SPF) breeding colony and acclimated for one week under controlled conditions (22 ± 1 °C, 12 h light/12 h dark, food and water ad libitum). After overnight fasting, the animals were randomly distributed into four experimental cohorts (*n* = 6 per group): Sham, MCAO, sh-NC, and sh-AU020206. Transient focal cerebral ischemia was induced using the intraluminal filament middle cerebral artery occlusion/reperfusion (MCAO/R) technique [21,22,23]. In the Sham cohort, the right middle cerebral artery was visualized but not occluded, whereas mice assigned to the remaining groups underwent 60 min of occlusion followed by reperfusion. One week before surgery, animals in the sh-NC and sh-AU020206 groups received a single tail vein injection of 1 × 10^8^ TU/mL lentiviral particles encoding either scrambled shRNA or shRNA against lncRNA-AU020206 (GenePharma, Shanghai, China) diluted in sterile saline [24]. Neurological impairments were quantified 24 h after reperfusion using the modified neurological severity score (mNSS), which integrates the assessments of motor coordination, sensory perception, reflex integrity, and balance. The scores ranged from 0 (no functional deficit) to 18 (severe neurological dysfunction) [25].

### 2.2. Infarct Volume Assessment

Cerebral infarction was quantified using 2,3,5-triphenyltetrazolium chloride (TTC) staining. After neurological testing, mice were deeply anesthetized and perfused with ice-cold saline; brains were rapidly removed, chilled at −20 °C for 60 s to facilitate sectioning, and sliced coronally into four 1 mm thick sections with a brain matrix. Sections were incubated in 1.5% TTC (Sigma-Aldrich, St. Louis, MO, USA) at 37 °C for 20 min in the dark and then fixed in 4% paraformaldehyde overnight. Viable tissue appeared brick red, whereas the infarcted areas remained unstained. Digital images were analyzed using ImageJ software 1.50 (NIH, Bethesda, MD, USA), and the infarct volume was calculated as a percentage of the ipsilateral hemisphere, correcting for edema [26].

### 2.3. Haematoxylin–Eosin (HE) Staining

Paraffin-embedded coronal brain sections (4 µm) were deparaffinized in xylene (2 × 5 min) and rehydrated in distilled water using graded ethanol (100%, 95%, 80%, and 70%; 2 min each). The slides were then immersed in a haematoxylin solution (AWI0009a, Abiowell, Changsha, China) for 5 min, rinsed under running tap water for 2 min, and blued in pH 7.4 PBS (AWC0217, Abiowell, China) for 1 min. After a second brief rinse, the sections were counterstained with eosin Y (AWI0029a, Abiowell, China) for 90 s, washed in distilled water, and dehydrated using increasing alcohol concentrations (95% and 100%; 2 min each). Finally, the specimens were cleared in xylene (2 × 3 min) and coverslipped with a neutral resin. Morphological alterations were examined under a light microscope (×200).

### 2.4. Detection of Apoptosis Using TUNEL Staining

A 1 × Proteinase K working solution was prepared for each section by diluting 1 µL of the 100 × stock (39450-01-6, Aladdin, Shanghai, China) in 99 µL of 1 × PBS. After gently blotting off the excess equilibration buffer, 50 µL of TdT reaction mixture was applied to each slide and incubated in a humidified, light-protected chamber at 37 °C for 60 min to prevent desiccation. The slides were then rinsed three times with 1 × PBS (5 min each). The nuclei were counterstained with DAPI (AWC0292, Abiowell, China) at 37 °C for 10 min in the dark, followed by three additional PBS washes (5 min each) before mounting.

### 2.5. N2a Cell Culture and Oxygen Glucose Deprivation (OGD) Treatment

Mouse neuroblastoma N2a cells (ATCC, CCL-131) were used to model neuronal ischemia. Cultures were maintained at 37 °C under normoxic conditions (95% air/5% CO_2_) in high-glucose DMEM supplemented with 10% fetal bovine serum. For oxygen–glucose deprivation/reoxygenation (OGD/R), cells were rinsed twice with glucose-free DPBS and transferred to a modular incubator chamber flushed with 95% N_2_/5% CO_2_ (0% O_2_) at 37 °C for 5 h. Reoxygenation was initiated by replacing the DPBS with fresh complete medium and returning the plates to normoxia for 24 h. Parallel control cultures were kept under normoxic conditions for the entire period. To determine the AU020206/YTHDC2 axis, OGD/R-treated N2a cells were allotted to four groups (*n* = 3 independent wells per condition): sh-NC (transfected with a scrambled shRNA plasmid); sh-AU020206 (transfected with an shRNA construct targeting lncRNA AU020206); sh-AU020206 + oe-NC (co-transfected with sh-AU020206 and an empty overexpression vector); sh-AU020206 + oe-YTHDC2 (co-transfected with sh-AU020206 and a YTHDC2 overexpression plasmid). A separate series examined whether METTL3 could counteract AU020206 silencing. The cells were divided into oe-NC (empty vector), oe-METTL3 (METTL3 overexpression), oe-METTL3 + oe-NC (METTL3 plus empty vector), and oe-METTL3 + oe-AU020206 (co-overexpression of METTL3 and AU020206) cells. Plasmids (Abiowell, Changsha, China) were delivered with Lipofectamine™ 2000 reagent (11668-500, Thermo Fisher Scientific, Waltham, MA, USA) according to the manufacturer’s instructions [27].

### 2.6. Cell Viability Assessment Using CCK-8

Cell viability was quantified using the Cell Counting Kit-8 (CCK-8; Dojindo Laboratories, Kumamoto, Japan) according to the manufacturer’s instructions. Briefly, N2a cells from each experimental group were seeded in 96-well plates at a density of 5 × 10^3^ cells/well in six replicates and allowed to adhere overnight. Following OGD/R treatment and the indicated genetic manipulations, 10 µL of CCK-8 reagent was added to each well containing 100 µL of culture medium, and plates were incubated at 37 °C for 2 h in the dark. The absorbance at 450 nm (reference: 650 nm) was measured using a microplate reader (BioTek, Winooski, VT, USA).

### 2.7. Flow Cytometry

After the indicated treatments, N2a cells were gently detached using trypsin lacking EDTA, pelleted at 2000 rpm for 5 min, and washed twice in ice-cold PBS. Approximately 5 × 10^5^ cells were resuspended in 500 µL Annexin V binding buffer (A377916, Aladdin, Shanghai, China). To each suspension, 5 µL Annexin V–FITC (KGA108, KeyGen, Beijing, China) and 5 µL propidium iodide were added, followed by a 15 min incubation at room temperature in the dark. Samples were analyzed immediately using a flow cytometer (A00-1-1102, Beckman Coulter, Inc., Brea, CA, USA) to quantify early and late apoptotic populations.

### 2.8. Enzyme-Linked Immunosorbent Assay (ELISA)

Commercial ELISA kits specific for TNF-α (CSB-E04740h), IL-1β (CSB-E08053h), IL-6 (CSB-E04638h), and IL-10 (CSB-E04593h) were purchased from Wuhan Huamei Biotechnology Co., Ltd. (Wuhan, China). All reagents were prepared in strict accordance with the manufacturers’ protocols. Standards and diluted samples were dispensed into the appropriate wells of precoated 96-well plates. Following incubation, the supernatant was decanted, and the plates were gently tapped on absorbent paper to remove residual fluid. Without an intermediate soak, each well was rinsed with 200 µL of wash buffer for 2 min; this wash step was repeated once, after which the plates were again blotted dry. The chromogenic reaction was terminated as directed, and within 5 min, the absorbance at 450 nm was recorded using a microplate spectrophotometer (MB-530; HEALES, Shenzhen, China).

### 2.9. RNA Pull-Down and RNA Immunoprecipitation (RIP)

For each sample, two microcentrifuge tubes were prepared and designated “IP” and “IgG” (normal rabbit IgG, 30000-0-AP, Proteintech, Wuhan, China). Clarified lysate (900 µL) was added to the IP tube containing pre-washed magnetic beads in RIP immunoprecipitation buffer, and the mixture was gently rotated overnight at 4 °C. As an input control, 10 µL of the original lysate was combined with an equal volume of 2 × SDS loading buffer and denatured at 95 °C for 5 min. After incubation, the bead–antibody complexes were placed on a magnetic stand, the supernatant was discarded, and each tube was rinsed once with 500 µL RIP wash buffer followed by a second wash with 250 µL buffer, gently mixing each time. The RNA recovered from the beads was reverse transcribed and the resulting cDNA was immediately used for conventional PCR or quantitative real-time PCR analyses.

### 2.10. Methylated RNA Immunoprecipitation (MeRIP)

The assay plate was gently rocked several times to ensure complete coverage of the well surface. For single-point controls, 2 µL of the positive control (0.5 ng/µL) was dispensed into the designated wells, yielding final RNA amounts of 0.02, 0.1, 0.2, 0.4, and 1 ng per well. To guarantee optimal binding efficiency, the volume of each experimental RNA sample did not exceed 8 µL. Colour development was monitored throughout the incubation period. Following the reaction, 100 µL blocking solution was added to each well to terminate enzymatic activity. The addition of the stop solution elicited a yellow colour, and the absorbance at 450 nm was recorded within 2–10 min using a microplate reader.

### 2.11. RNA Stability Assay

RNA turnover was assessed in N2a neuroblastoma cells by pharmacological blockade of transcription. Confluent cultures were switched to fresh complete medium containing actinomycin D (5 µg/mL; Sigma-Aldrich Corporation, St. Louis, MO, USA) and harvested at 0, 1, 2, 4, and 6 h post-addition. At each time point, the cells were rinsed twice with ice-cold PBS, lysed in TRIzol reagent, and total RNA was purified as described above. One microgram of RNA was reverse-transcribed with gene-specific primers, and the transcript levels of AU020206, SLC7A11, and GAPDH were quantified using qRT-PCR (ABI 7900HT). Relative RNA levels were calculated using the 2^−ΔΔCt^ method (ΔCt = Ct_target − Ct_GAPDH) and normalized to the 0 h value. First-order degradation constants (k) and half-lives (t_1/2_ = ln2/k) were obtained using linear regression of ln(relative abundance) versus time. All conditions were analyzed in triplicate in three independent experiments.

### 2.12. Quantitative Real-Time PCR (qRT-PCR)

Total RNA was extracted from C57BL/6 mouse cortices and N2a neuroblastoma cells using TRIzol reagent (Invitrogen, Carlsbad, CA, USA) following the supplier’s protocol. One microgram of purified RNA was reverse-transcribed with the TaqMan™ MicroRNA/mRNA Reverse-Transcription Kit (Thermo Fisher Scientific, Waltham, MA, USA) using gene-specific stem-loop primers for miRNAs or oligo-dT/random hexamer primers for mRNAs/lncRNAs. Quantitative PCR was performed on an ABI Prism 7900HT real-time system (Applied Biosystems, Foster City, CA, USA) with All-in-One™ qPCR Mix (GeneCopoeia, Inc., Rockville, MD, USA). Cycling parameters were: 95 °C for 5 min (initial denaturation) followed by 40 cycles of 95 °C for 15 s, 60 °C for 30 s and 72 °C for 30 s, with a final extension at 72 °C for 5 min. Relative transcript levels were calculated by the 2^−ΔΔCt^ method, normalizing lncRNA and mRNA targets to GAPDH. Tfrc, Acsl4, Slc7a11, and Gpx4 were chosen for analysis because they are well-established regulators of ferroptosis and oxidative stress: Tfrc mediates iron uptake and contributes to iron overload, Acsl4 facilitates lipid peroxidation, Slc7a11 functions as a cystine/glutamate antiporter essential for glutathione synthesis, and Gpx4 is a central antioxidant enzyme that prevents lipid peroxidation [28]. These genes were therefore included to assess whether AU020206 might exert regulatory effects through ferroptosis- and apoptosis-related targets. All reactions were performed in triplicate. The primer sequences used were in Table 1.

### 2.13. Western Blot Analysis

Protein extracts were prepared from ischemic cortex samples and cultured in N2a cells using ice-cold RIPA buffer (Haimen Biotechnology, Suzhou, China) supplemented with protease-phosphatase inhibitors. The homogenates were kept on ice for 30 min and cleared using centrifugation (12,000× *g*, 15 min, 4 °C), and the supernatants were assayed for protein concentration using a BCA kit (Thermo Fisher Scientific, USA). Equal aliquots of total protein (25–40 µg per lane) were separated on 10–12% SDS-PAGE gels (Sigma-Aldrich, Inc., St. Louis, MO, USA) and electroblotted onto nitrocellulose membranes (Pall Corporation, Port Washington, NY, USA). Membranes were blocked for 2 h in 5% non-fat dry milk prepared in TBST (20 mM Tris-HCl, 150 mM NaCl, 0.1% Tween-20, pH 7.4) and incubated overnight at 4 °C with the following primary antibodies: METTL3 (1:1000, Cell Signalling Technology, Danvers, MA, USA), YTHDC2 (1:1000, Proteintech, Wuhan, China), SLC7A11 (1:800, Abcam, Cambridge, UK), GPX4 (1:1000, Abcam) and GAPDH (1:5000, Proteintech). After three TBST washes, the membranes were incubated with horseradish peroxidase-conjugated goat anti-rabbit or anti-mouse IgG secondary antibodies (1:5000; Jackson ImmunoResearch, West Grove, PA, USA) for 1 h at room temperature. Bands were visualized with enhanced chemiluminescence reagent (Pierce ECL, Rockford, IL, USA) and captured on a ChemiDoc™ XRS+ imaging system (Bio-Rad Laboratories, Hercules, CA, USA). Densitometric analysis was performed with Quantity One software4.6.2, and protein levels were normalized to β-actin. Each experiment was independently repeated at least three times.

### 2.14. Statistical Information

All results are presented as mean ± standard deviation (SD). Intergroup differences were evaluated using one-way analysis of variance (ANOVA), followed, where appropriate, by Tukey’s post hoc test for multiple comparisons. Prior to performing ANOVA, the assumptions of normality and homogeneity of variance were verified using the Shapiro–Wilk test and Levene’s test, respectively. Only when these assumptions were met were parametric analyses applied; otherwise, appropriate non-parametric alternatives were considered. Exact sample sizes for each experiment are indicated in the corresponding figure legends. All statistical analyses were performed using SPSS v27.0 (IBM Corp., Armonk, NY, USA). A two-tailed *p* < 0.05 was taken as the criterion for statistical significance. Each experiment was conducted independently at least three times to ensure reproducibility and analytical robustness.

## 3. Results

### 3.1. Increased Expression of lncRNA-AU020206 Is Closely Associated with Ferroptosis Induced by I/R In Vivo and In Vitro

**Figure 1:**
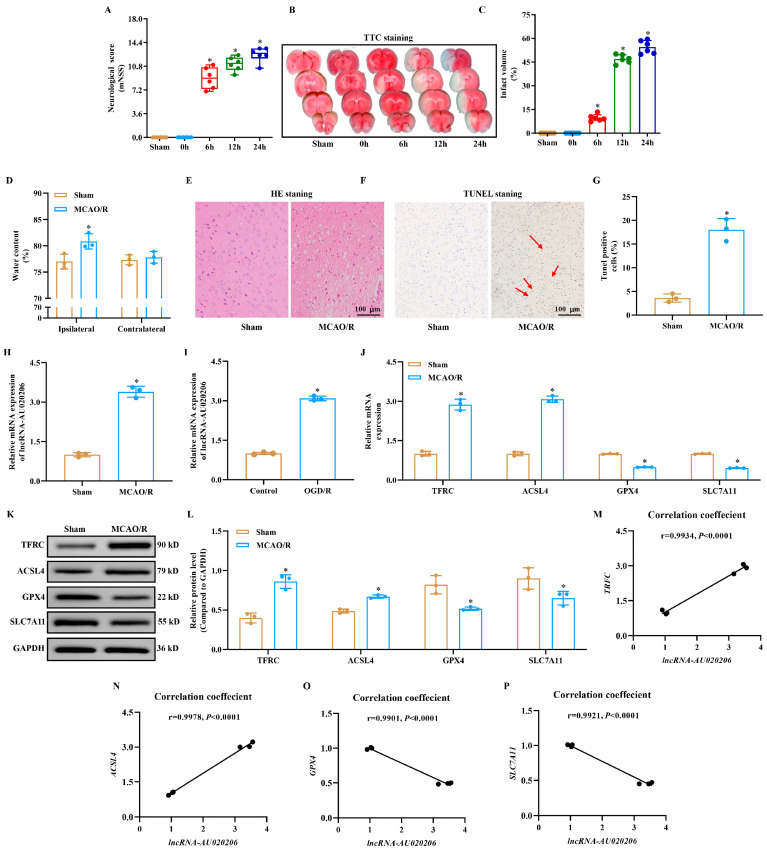
Increased expression of lncRNA-AU020206 is closely associated with ferroptosis induced by cerebral ischemia/reperfusion (I/R) in vivo and in vitro. (**A**) Modified neurological severity scores (mNSS) of mice subjected to sham surgery or middle cerebral artery occlusion/reperfusion (MCAO/R) at indicated reperfusion time points (0 h, 6 h, 12 h, 24 h). (**B**) Representative TTC-stained brain slices from mice at different reperfusion intervals. (**C**) Quantification of infarct volume across groups. (**D**) Brain water content in ipsilateral and contralateral hemispheres at 24 h post-reperfusion. (**E**) Representative hematoxylin-eosin (HE) staining of cortex and (**F**) TUNEL staining for apoptotic cells in brain tissue from sham and MCAO/R mice. Sham shows few TUNEL-positive cells, whereas MCAO/R shows abundant apoptotic nuclei (**arrows**). Scale bar = 100 μm. (**G**) Quantification of TUNEL-positive cells per field. (**H**) Relative expression of lncRNA-AU020206 in mouse brains after MCAO/R assessed by qRT-PCR. (**I**) lncRNA-AU020206 expression in N2a cells after oxygen-glucose deprivation/reoxygenation (OGD/R). (**J**) qRT-PCR analysis of ferroptosis-related mRNA expression (TFRC, ACSL4, GPX4, SLC7A11) in brains of sham and MCAO/R mice. (**K**) Representative Western blot for ferroptosis markers and GAPDH in brain tissues (original images can be found in Supplementary Materials). (**L**) Quantification of protein expression relative to GAPDH. (**M**–**P**) Correlation analysis between lncRNA-AU020206 and the mRNA levels of TFRC, ACSL4, GPX4, and SLC7A11. Data are shown as mean ± SD. * *p* < 0.05 vs. Sham.

By establishing an MCAO/R mouse model, we systematically evaluated the pathological alterations in brain tissue following ischemia–reperfusion injury. Compared to the sham group, mice in the MCAO/R group exhibited substantially increased neurological deficit scores (Figure 1A), with markedly enlarged infarct volumes, and elevated brain water content (Figure 1B–D). HE staining indicated that the cortical structures were well preserved, and the cellular architecture was intact in the sham group. In contrast, the MCAO/R group displayed liquefactive necrosis, disrupted cellular arrangement, pronounced perilesional edema, nuclear pyknosis or karyolysis, and inflammatory cell infiltration (Figure 1E). Consistently, TUNEL staining demonstrated a marked increase in apoptotic cells in the brains of MCAO/R mice (Figure 1F). At the molecular level, lncRNA-AU020206 expression was markedly upregulated in the brains of MCAO/R mice compared to sham controls. This upregulation was also observed in OGD/R-challenged N2a cells, which reflected the in vivo results (Figure 1H,I). Furthermore, both the mRNA and protein levels of TFRC and ACSL4 were elevated, whereas SLC7A11 and GPX4 expression was markedly decreased in the MCAO/R group (Figure 1J,K), indicating the induction of ferroptosis after ischemia/reperfusion (I/R) injury. Correlation analysis indicated that lncRNA-AU020206 expression was positively correlated with TFRC and ACSL4 but negatively correlated with SLC7A11 and GPX4 (Figure 1M–P). Collectively, these results suggest that the abnormal expression of lncRNA-AU020206 and ferroptosis-related genes is closely associated in vivo and in vitro, highlighting the potential role of lncRNA-AU020206 in I/R-induced ferroptosis.

### 3.2. Silencing lncRNA-AU020206 Markedly Attenuates OGD/R-Induced Apoptosis, Inflammation, and Ferroptosis In Vitro

**Figure 2:**
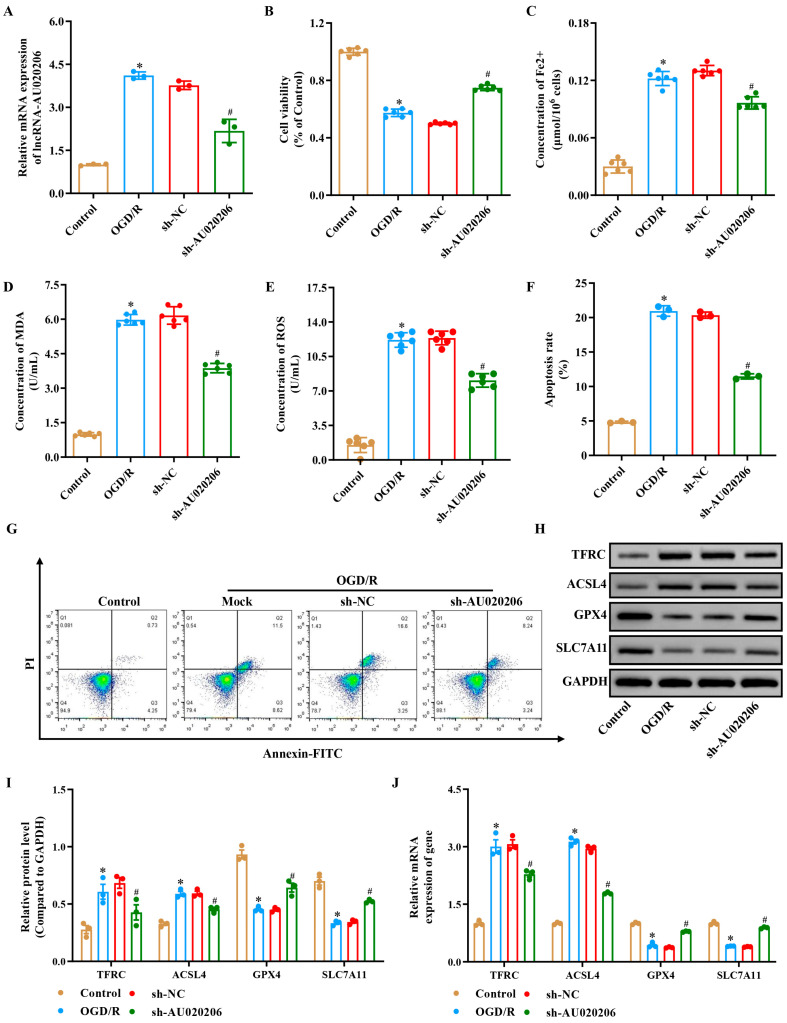
Silencing lncRNA-AU020206 attenuates OGD/R-induced apoptosis, oxidative stress, and ferroptosis in N2a cells. (**A**) Relative lncRNA-AU020206 expression following Control, OGD/R, sh-NC, or sh-AU020206 transfection. (**B**) Cell viability assessed by CCK-8 assay. (**C**) Intracellular Fe^2+^ concentration. (**D**) Malondialdehyde (MDA) levels. (**E**) Reactive oxygen species (ROS) concentration. (**F**) Apoptosis rate detected by flow cytometry. (**G**) Representative Annexin V–FITC/PI flow cytometry plots for apoptosis analysis. (**H**,**I**) Western blot for TFRC, ACSL4, GPX4, and SLC7A11 expression in N2a cells. (**J**) Quantification of relative mRNA levels (normalized to GAPDH) under different conditions. Data are shown as mean ± SD. * *p* < 0.05 vs. control; # *p* < 0.05 vs. sh-NC.

To elucidate the functional role of lncRNA-AU020206 in I/R injury, we constructed an OGD/R model using N2a cells and silenced lncRNA-AU020206 expression. N2a cells, a well-characterized mouse neuroblastoma line, were chosen because they are widely used as an in vitro neuronal model owing to their reproducible growth characteristics and high sensitivity to OGD/R challenge, which makes them suitable for mimicking ischemia/reperfusion injury in vitro [29]. Following OGD/R exposure, lncRNA-AU020206 levels were substantially elevated, whereas sh-lncRNAAU020206 transfection effectively suppressed its expression, confirming the efficiency of gene silencing (Figure 2A). OGD/R exposure led to a pronounced reduction in cell viability (Figure 2B), accompanied by elevated intracellular Fe^2+^ concentrations (Figure 2C), lipid peroxidation marker MDA levels (Figure 2D), and reactive oxygen species (ROS) production (Figure 2E). In contrast, these alterations were significantly reversed in the sh-AU020206 group, indicating a protective effect of AU020206 knockdown against OGD/R-induced oxidative stress and lipid damage. Moreover, flow cytometry analysis further revealed a significantly increased apoptosis rate in OGD/R-treated cells, which was markedly reduced following AU020206 silencing (Figure 2F,G). With respect to ferroptosis, OGD/R exposure led to increased mRNA and protein levels of TFRC and ACSL4 and decreased SLC7A11 and GPX4 expression. Silencing lncRNA-AU020206 reversed these changes, as evidenced by reduced TFRC and ACSL4 and upregulated SLC7A11 and GPX4 expression (Figure 2H–J). Consistently, measurement of Fe^2+^ content indicated a marked reduction in N2a cell Fe^2+^ in the sh-AU020206 compared to the sh-NC group (Figure 2C). Collectively, these findings demonstrate that silencing lncRNA-AU020206 mitigated OGD/R-induced N2a cell injury, suppressed apoptosis and modulated the ferroptosis pathway.

### 3.3. Silencing lncRNA-AU020206 Alleviates MCAO/R-Induced Injury In Vivo

**Figure 3:**
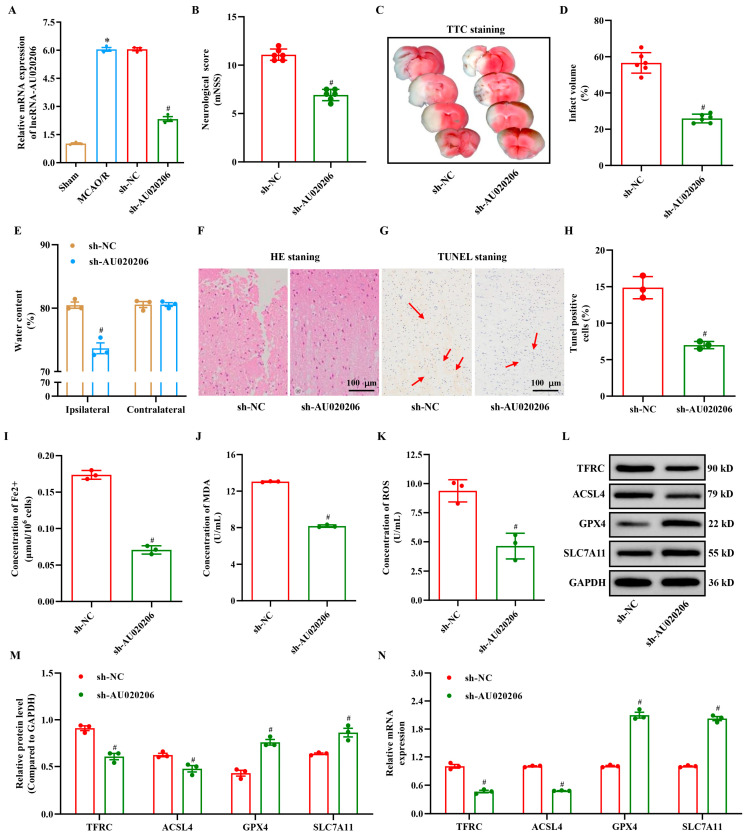
Silencing lncRNA-AU020206 alleviates MCAO/R-induced brain injury and ferroptosis in vivo. (**A**) Relative lncRNA-AU020206 expression in the brains of sham, MCAO/R, sh-NC, and sh-AU020206 mice. (**B**) Neurological deficit scores at 24 h post-reperfusion. (**C**) Representative TTC-stained brain sections and (**D**) infarct volume quantification. (**E**) Brain water content in ipsilateral and contralateral hemispheres. (**F**) Representative HE staining and (**G**) TUNEL staining of cortical sections 24 h after MCAO/R. The sh-NC group shows numerous TUNEL-positive apoptotic nuclei (**red arrows**), whereas the sh-AU020206 group shows fewer TUNEL-positive cells. Scale bar = 100 μm. (**H**) Quantification of TUNEL-positive cells per field. (**I**–**K**) Quantification of Fe^2+^, MDA, and ROS levels in brain tissues. (**L**) Western blot analysis of TFRC, ACSL4, GPX4, and SLC7A11 expression. (**M**) Densitometric analysis of protein bands (relative to GAPDH). (**N**) Relative mRNA levels of ferroptosis-related genes determined by qRT-PCR. Data are shown as mean ± SD. * *p* < 0.05 vs. Sham; # *p* < 0.05 vs. sh-NC.

Our results from the N2a cell model demonstrated that silencing lncRNA-AU020206 reduced cellular injury and ferroptosis. To validate these findings in vivo, we assessed the effects of the lncRNA-AU020206 knockdown in a mouse MCAO/R model (Figure 3A). Mice treated with sh-lncRNA-AU020206 exhibited substantially improved neurological scores compared to the sh-NC group (Figure 3B). TTC staining indicated that the infarct volume and brain water content were markedly reduced in the sh-AU020206 compared to the sh-NC group (Figure 3C–E). Histopathological analysis further supported these findings; widespread necrosis and inflammatory cell infiltration were observed in the ischemic cortex of sh-NC mice, whereas the sh-AU020206 group showed reduced necrosis (Figure 3F), with fewer apoptotic cells (Figure 3G,H). In addition, the expression levels of the ferroptosis-related genes, *SLC7A11* and *GPX4* expression (both mRNA and protein) were substantially increased, whereas TFRC and ACSL4 levels (both mRNA and protein) were notably decreased in the sh-AU020206 group (Figure 3L–N). Consistently, the measurement of Fe^2+^ concentrations (Figure 3I), MDA levels (Figure 3J), and ROS production (Figure 3K) indicated a marked reduction in brain tissue Fe^2+^ in the sh-AU020206 compared to the sh-NC group. Collectively, these results indicate that silencing lncRNA-AU020206 can alleviate brain injury in the MCAO/R mouse model by regulating ferroptosis, thereby improving neurological outcomes and reducing infarct size.

### 3.4. lncRNA-AU020206 and YTHDC2 Synergistically Regulate Apoptosis, Inflammation, and Ferroptosis in OGD/R-Induced N2a Cells

**Figure 4:**
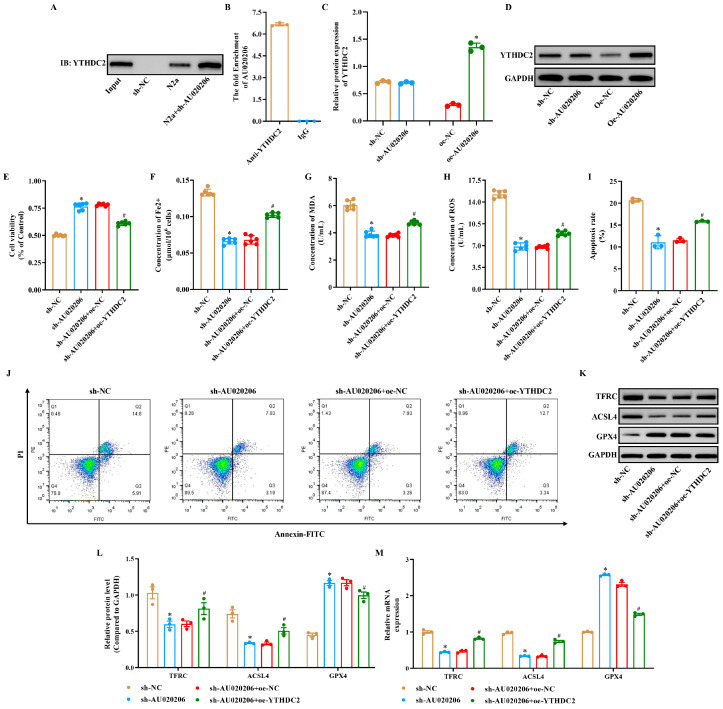
lncRNA-AU020206 interacts with YTHDC2 to modulate apoptosis and ferroptosis in OGD/R-injured N2a cells. (**A**,**B**) RNA pull-down and RIP analysis showing interaction between lncRNA-AU020206 and YTHDC2. (**C**,**D**) Protein expression of YTHDC2 by Western blot. (**E**) Cell viability after indicated genetic manipulations. (**F**–**H**) Quantification of Fe^2+^, MDA, and ROS in N2a cells. (**I**) Apoptotic rate assessed by flow cytometry. (**J**) Representative flow cytometry plots for apoptosis analysis. (**K**) Western blot for TFRC, ACSL4, GPX4, and SLC7A11 expression. (**L**) Quantification of protein expression relative to GAPDH. (**M**) qRT-PCR analysis of ferroptosis-related mRNAs. Data are shown as mean ± SD. * *p* < 0.05 vs. sh-NC; # *p* < 0.05 vs. sh-AU020206+oe-NC.

Previous studies and our research have demonstrated that silencing lncRNA-AU020206 alleviates brain tissue injury and inhibits ferroptosis [30]. Recent studies have revealed that m6A methylation regulates not only protein-coding transcripts but also non-coding RNAs, including lncRNAs, by affecting their stability, subcellular localization, and binding affinity with RNA-binding proteins (RBPs) [31]. Notably, the presence of consensus DRACH motifs (D = A/G/U, R = A/G, H = A/C/U) within lncRNA sequences often indicates potential sites for m6A modification. In silico analysis using SRAMP and RMBase v2.0 databases revealed multiple high-confidence m6A sites within AU020206 transcript, suggesting that it may undergo post-transcriptional regulation via METTL3-mediated m6A deposition. Consistently, StarBase v2.0 prediction also indicated the presence of potential binding sites between AU020206 and YTHDC2. Furthermore, YTHDC2, an m6A reader with helicase activity, is known to bind to m6A-modified RNAs and mediate their degradation or translation in a context-dependent manner [32]. These observations prompted us to investigate whether AU020206 directly interacts with YTHDC2 in an m6A-dependent manner in the setting of cerebral I/R injury. To elucidate the regulatory mechanism, we investigated the interaction between lncRNA-AU020206 and the m6A reader protein, YTHDC2. Our bioinformatic analysis predicted a potential binding site between lncRNA-AU020206 and YTHDC2, which was subsequently confirmed by RNA pull-down and Western blot assays (Figure 4A). RIP assays indicated a marked enrichment of lncRNA-AU020206 in the YTHDC2-bound fraction (Figure 4B). Although there was no marked difference in YTHDC2 expression between the sh-NC and sh-lncRNA-AU020206 groups, YTHDC2 expression was markedly elevated in the oe-YTHDC2 group (Figure 4C,D). Functionally, silencing lncRNA-AU020206 markedly A can significantly attenuate the reduction in cell viability and the increase in apoptosis induced by OGD/R in N2a cells, whereas combined treatment with YTHDC2 overexpression (sh-AU020206 + oe-YTHDC2 group) restored the cell viability and apoptotic rate, indicating that YTHDC2 overexpression reversed the mitigation of cellular injury and enhancement of anti-apoptotic effect of lncRNA-AU020206 silencing (Figure 4E,I,J). In contrast, silencing of lncRNA-AU020206 increased SLC7A11 and GPX4 expression, while reducing TFRC and ACSL4 levels; these effects were reversed upon YTHDC2 overexpression (Figure 4K–M). Furthermore, intracellular Fe^2+^ content (Figure 4F), MDA levels (Figure 4G), and ROS production (Figure 4H) were decreased in the sh-AU020206 group, whereas it was substantially increased in the sh-AU020206 + oe-YTHDC2 group. Collectively, these results indicate that YTHDC2 counteracts the protective effects of lncRNA-AU020206 silencing in OGD/R-injured N2a cells by regulating I/R-induced ferroptosis.

### 3.5. lncRNA-AU020206 and YTHDC2 Coordinately Regulate SLC7A11 Expression

**Figure 5:**
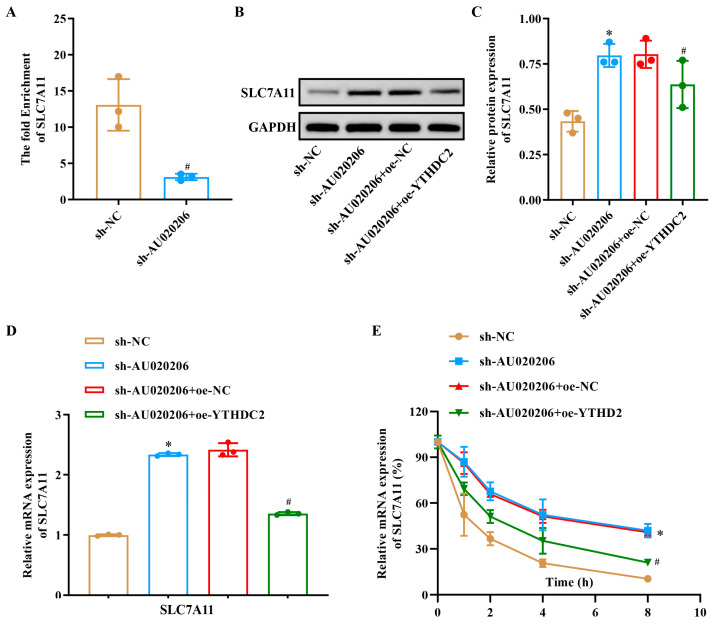
YTHDC2 modulates SLC7A11 stability and expression in concert with lncRNA-AU020206 in OGD/R-injured N2a cells. (**A**) RIP enrichment of SLC7A11 following lncRNA-AU020206 silencing or YTHDC2 overexpression. (**B**) Western blot for SLC7A11 and GAPDH protein expression. (**C**) Quantification of relative SLC7A11 protein levels. (**D**) Relative SLC7A11 mRNA expression assessed by qRT-PCR in indicated groups. (**E**) Time-course analysis of SLC7A11 mRNA stability in actinomycin D-treated N2a cells. Data are shown as mean ± SD. * *p* < 0.05 vs. sh-NC; # *p* < 0.05 vs. sh-AU020206+oe-NC.

Our previous experiments have confirmed that lncRNA-AU020206 and YTHDC2 can work together to exert their effects. Since YTHDC2 is an m6A “reader” protein known to modulate the stability and translation of m6A-modified RNAs, we hypothesized that AU020206 might act as an m6A-modified scaffold interacting with YTHDC2, thereby influencing the post-transcriptional regulation of SLC7A11, a key ferroptosis-related gene. To investigate whether the interaction between lncRNA-AU020206 and YTHDC2 affected SLC7A11 expression, we performed RIP assays to assess the enrichment of SLC7A11 following lncRNA-AU020206 overexpression. SLC7A11 enrichment was substantially reduced in the sh-AU020206 compared to the oe-NC group (Figure 5A), indicating that lncRNA-AU020206 was involved in the regulation of SLC7A11 expression. Furthermore, both the mRNA and protein levels of SLC7A11 were higher in the sh-AU020206 than in the sh-NC group. However, in the sh-AU020206+oe-YTHDC2 group, SLC7A11 mRNA and protein expression was decreased compared to that in the sh-AU020206+oe-NC group (Figure 5B–D). To validate these findings, we monitored SLC7A11 expression in actinomycin-D-treated N2a cells over time. The SLC7A11 expression in the sh-AU020206+oe-YTHDC2 group progressively decreased, approaching levels similar to those in the control group (Figure 5E). Collectively, these results suggest that both lncRNA-AU020206 and YTHDC2 regulate SLC7A11 expression; silencing lncRNA-AU020206 promotes SLC7A11 expression, whereas overexpression of YTHDC2 exerts an inhibitory effect.

### 3.6. METTL3-Mediated m6A Modification of lncRNA-AU020206 Suppresses Apoptosis, Inflammation, and Ferroptosis Induced by Cerebral Ischemia–Reperfusion Injury

**Figure 6:**
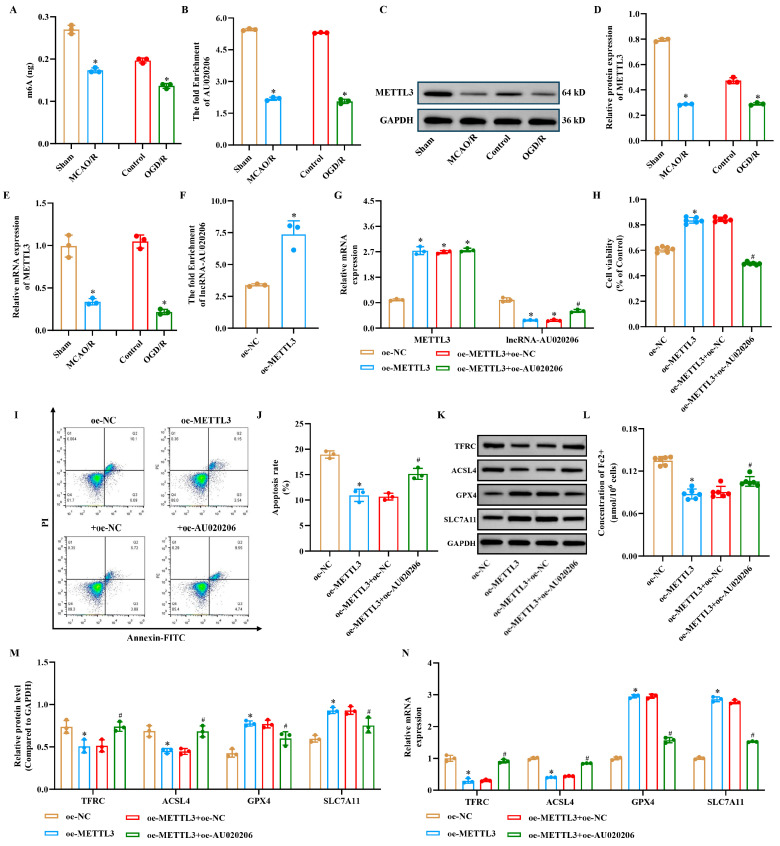
METTL3-mediated m6A modification of lncRNA-AU020206 suppresses apoptosis and ferroptosis in ischemic conditions. (**A**) m6A methylation levels in brain tissues of sham and MCAO/R mice, and in control and OGD/R-treated N2a cells. (**B**,**C**) Enrichment and protein expression of METTL3 in indicated groups. (**D**,**E**) Relative expression of m6A-modified lncRNA-AU020206 and SLC7A11 assessed by qRT-PCR. (**F**) MeRIP-qPCR and RIP assays showing m6A modification and METTL3 binding to AU020206. (**G**) Relative mRNA expression of METTL3 and lncRNA-AU020206 after overexpression. (**H**) Cell viability after genetic manipulation. (**I**,**J**) Apoptosis rate assessed by flow cytometry and representative plots. (**K**) Western blot of ferroptosis markers. (**L**) Fe^2+^ content in N2a cells. (**M**,**N**) Quantification of protein and mRNA levels of TFRC, ACSL4, GPX4, and SLC7A11. Data are shown as mean ± SD. * *p* < 0.05 vs. oe-NC; # *p* < 0.05 vs. oe-METTL3+oe-NC.

To elucidate the role of METTL3 in m6A modification during cerebral ischemia–reperfusion injury, we assessed m6A levels in the brains of Sham and MCAO/R mice. Overall, m6A methylation was substantially reduced in the MCAO/R compared to that in the sham group. Similarly, m6A levels decreased in the OGD/R group relative to the control in vitro, which was consistent with the in vivo findings (Figure 6A). Furthermore, m6A modification, mRNA and protein expression of lncRNA-AU020206 was markedly decreased in both the MCAO/R and OGD/R groups (Figure 6B–E). RIP assays indicated that METTL3 expression was downregulated in the MCAO/R group, whereas METTL3 overexpression markedly increased m6A modification of lncRNA-AU020206 (Figure 6F). To clarify the functions of METTL3 and AU020206, we established overexpression models of both genes. In the oe-METTL3 + oe-AU020206 group, METTL3 levels remained unchanged, whereas AU020206 expression was elevated, confirming the success of the overexpression constructs (Figure 6G). Functionally, overexpression of METTL3 markedly reduced apoptosis in N2a cells; however, in the oe-METTL3+oe-AU020206 group, the cell viability was markedly lower and apoptotic rate was higher than that in the oe-METTL3+oe-NC group, suggesting that AU020206 overexpression partially reversed the anti-injury effect of METTL3 overexpression (Figure 6H–J). SLC7A11 and GPX4 mRNA and protein expression were upregulated, whereas TFRC and ACSL4 were downregulated in the oe-METTL3 group; these changes were reversed in the oe-METTL3+oe-AU020206 group (Figure 6K,M,N). Similarly, Fe^2+^ content was reduced in the oe-METTL3 group and increased upon AU020206 overexpression (Figure 6L). Taken together, these findings demonstrate that METTL3 overexpression facilitates m6A modification of lncRNA-AU020206 and markedly suppresses apoptosis, inflammation, and ferroptosis in N2a cells, an effect that can be counteracted by AU020206 overexpression.

## 4. Discussion

This study, for the first time, elucidated the intricate mechanism by which METTL3-driven m6A methylation modulates ferroptosis in cerebral I/R injury, primarily through the suppression of lncRNA-AU020206 expression and subsequent regulation of SLC7A11 stability. Both the in vivo MCAO/R mouse model and in vitro OGD/R-treated N2a cells exhibited pronounced upregulation of lncRNA-AU020206, which was closely associated with the aberrant expression of ferroptosis-related markers, including increased TFRC and ACSL4, and decreased GPX4 and SLC7A11. Functional experiments demonstrated that silencing of AU020206 substantially ameliorated neuronal apoptosis, reduced inflammatory cytokine secretion, and attenuated ferroptotic cell death, ultimately improving neurological outcomes, reducing infarct volume, and limiting histopathological damage. Mechanistically, the data indicated that METTL3 catalyzed the m6A modification of AU020206, thereby promoting its degradation and downregulating its expression. Furthermore, AU020206 silencing disrupted its interaction with the m6A reader protein, YTHDC2, resulting in decreased SLC7A11 mRNA degradation and elevated SLC7A11 protein levels, which protected against I/R-induced ferroptosis. Genetic interventions targeting this pathway, such as the knockdown of YTHDC2 or AU020206, were found to substantially ceased the neuroprotective effects mediated by METTL3, underscoring the central role of the METTL3/lncRNA-AU020206/YTHDC2/SLC7A11 signalling axis in the context of I/R injury. Collectively, these findings not only expand our current understanding of the m6A epitranscriptomic landscape and lncRNA regulatory networks in neurological disorders but also highlight promising molecular targets for developing novel therapeutic strategies against ischemic brain injury.

In recent years, lncRNAs have emerged as pivotal non-coding transcripts that orchestrate a wide array of biological processes and are critically involved in the pathogenesis of diverse diseases [33,34], especially within the central nervous system [35]. Compelling evidence has indicated that lncRNAs exert multidimensional regulatory effects on neuronal apoptosis, inflammatory responses, and ferroptosis following cerebral ischemic injury [36,37,38]. The lncRNA-AU020206 has been identified as a novel therapeutic target in atherosclerosis [39], with high expression observed in both hypoxic–ischemic brain tissue and astrocytes. Although previous studies have shown that silencing lncRNA-AU020206 can partially attenuate brain injury by inhibiting apoptosis [30], its precise role in the regulation of ferroptosis and neuroprotection remains unclear. In the current study, we provide the first evidence that lncRNA-AU020206 is markedly upregulated under I/R conditions and exhibits strong negative correlation with key ferroptosis-related molecules, including ACSL4, TFRC, GPX4, and SLC7A11. AU020206 silencing not only normalized the aberrant expression of ferroptosis markers but also substantially mitigated neuronal apoptosis and suppressed inflammatory cytokine release, thereby conferring broad neuroprotection. These effects closely resemble the biological functions of other brain injury-associated lncRNAs, such as MEG3 and PVT1 [40,41]. Furthermore, the data indicate that AU020206 interacts with the m6A reader protein, YTHDC2, to modulate downstream mRNA stability, suggesting that AU020206 functions as both a competing endogenous RNA (ceRNA) and an m6A-dependent scaffold RNA, facilitating the assembly of protein–RNA complexes and enhancing post-transcriptional regulation [42]. This dual regulatory mechanism substantially enriches our conceptual framework of lncRNA action in brain injury. Thus, AU020206 may act as both an upstream modulator of transcription and a downstream stabilizer of target mRNAs in I/R injury, highlighting its value as a promising molecular target for future therapeutic interventions in ischemic brain disorders.

The m6A is one of the most abundant internal RNA modifications in mammals, and plays a fundamental role in neural development, metabolic adaptation, and injury repair. The m6A expression is substantially higher in the brain than in other tissues, emphasizing its unique tissue-specific regulatory capacity in the central nervous system. Accumulating evidence indicates that m6A modifications modulate neuronal apoptosis and neuroinflammatory responses in cerebral ischemia by regulating mRNA stability, alternative splicing, and translational efficiency [43,44]. METTL3, a pivotal methyltransferase, is widely recognized for its critical involvement in m6A methylation and signalling regulation in various neurological diseases [45]. The current study demonstrated that ischemia/reperfusion injury induced the upregulation of YTHDC2, in line with previously observed changes in YTHDF1 and YTHDF2 after stroke. Consistently, the data confirm that m6A methylation not only influences gene sorting into stress granules, but also directly impacts the neuroprotective mechanisms following cerebral I/R injury [46]. In both the MCAO rat model and OGD/R-treated N2a cells, a marked decrease in m6A levels was observed. Further investigation indicated that METTL3 overexpression enhanced the m6A modification of lncRNA AU020206, promoted its degradation and reduced its expression. This effect, mediated by the AU020206/YTHDC2 complex, stabilized SLC7A11 mRNA, thereby mitigating ferroptosis and cellular injury. Although YTHDC2 has previously been implicated in mRNA degradation within the reproductive system [47], our findings indicate, for the first time, its regulatory role in ferroptosis during cerebral I/R injury. This mechanism is reminiscent of prior observations regarding METTL3-mediated stabilization of BATF and SNHG8 mRNAs [11,48], but the current study extends such regulatory paradigms to the level of lncRNAs. The overexpression of YTHDC2 reversed the neuroprotective effects conferred by AU020206 silencing, highlighting the indispensable role of this m6A reader in the regulatory axis. Collectively, these findings illustrate that METTL3 and YTHDC2, through m6A-dependent post-transcriptional regulation, constitute a novel “epitranscriptomic-safeguard” system in cerebral I/R injury, offering a new theoretical basis and a promising molecular target for neuroprotective therapies.

SLC7A11, as the core subunit of the system Xc^−^ antiporter, is responsible for the uptake of extracellular cystine and the synthesis of glutathione (GSH), serving as a central molecule in defending against ferroptosis-induced neuronal injury [49]. GSH acts as a major intracellular antioxidant and sustains the enzymatic activity of GPX4, which directly reduces lipid hydroperoxides, thus preventing ferroptosis. GPX4 is recognized as a principal inhibitor of ferroptotic cell death, playing a crucial role in maintaining neuronal integrity under pathological conditions [18,50]. In the context of cerebral I/R injury, ferroptosis is a key mechanism underlying neuronal loss and subsequent neurological dysfunction [51]. Downregulation of the SLC7A11/GPX4 pathway leads to GSH depletion, lipid ROS accumulation, and iron overload, all of which potentiate ferroptotic damage and exacerbate ischemic injury [52]. The current study indicates that in MCAO/R and OGD/R models, SLC7A11 expression was substantially reduced, accompanied by elevated Fe^2+^ levels, increased lipid ROS, and GPX4 depletion, indicating the activation of ferroptosis. Restoration of SLC7A11 expression substantially alleviated neuronal injury, and this effect was modulated by METTL3 or AU020206. Further mechanistic investigations indicated that AU020206, through its direct interaction with the m6A reader protein, YTHDC2, facilitated the degradation and destabilization of SLC7A11 mRNA, thereby reducing its steady-state levels and promoting ferroptotic susceptibility. This post-transcriptional regulatory effect enriches the current understanding of ferroptosis control, indicating that lncRNAs can fine-tune cell fate decisions not only through transcriptional interference but also via epitranscriptomic pathways [53]. In addition, the results suggest that this mechanism is distinct, yet complementary to the classical Nrf2/HO-1 pathway that activates SLC7A11 at the transcriptional level. By establishing this “transcription–epitranscription–translation” multi-layered regulatory axis, our data indicate how the interplay between METTL3, AU020206, and YTHDC2 precisely orchestrates ferroptosis during cerebral I/R injury. Considering that the SLC7A11/GPX4 axis has demonstrated therapeutic value in multiple disease models, including stroke, cancer, and sepsis, our study provides compelling evidence that targeting post-transcriptional regulators such as the METTL3/lncRNA-AU020206/YTHDC2 molecular network may yield novel and effective strategies for the prevention and treatment of ischemic brain injury.

## 5. Conclusions

**Figure 7:**
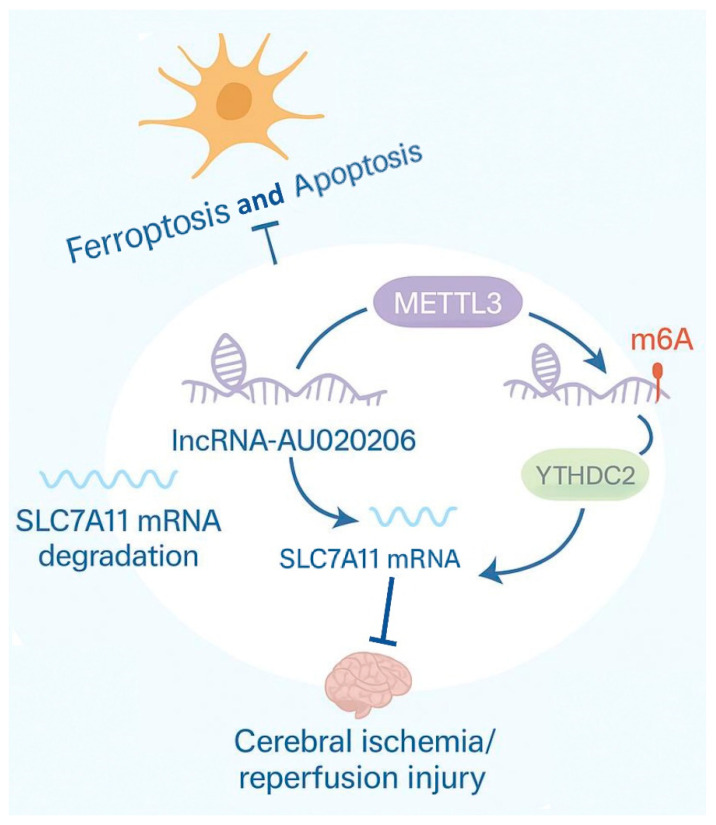
Schematic illustration of METTL3/lncRNA-AU020206/YTHDC2/SLC7A11 regulatory axis in cerebral ischemia/reperfusion injury. METTL3-mediated m6A modification of lncRNA-AU020206 enhances its degradation and decreases its interaction with YTHDC2, thereby stabilizing SLC7A11 mRNA and attenuating ferroptosis in neuronal cells following cerebral ischemia/reperfusion injury.

In summary, this study is the first to reveal that METTL3-driven m6A epigenetic remodelling by lncRNA-AU020206 acts as a critical brake on ferroptotic cell death in the context of cerebral I/R injury. METTL3-mediated m6A modification reduced lncRNA-AU020206 stability, thereby limiting its interaction with YTHDC2 and preventing excessive SLC7A11 mRNA degradation. This regulatory cascade ultimately preserved SLC7A11/GPX4 antioxidant capacity, restrained ferroptosis, and alleviated neuronal damage following I/R. These findings not only define a novel epitranscriptomic–lncRNA regulatory mechanism in ferroptosis but also posit that the METTL3/lncRNA-AU020206/YTHDC2/SLC7A11 axis is a promising target for therapeutic intervention in ischemic stroke (Figure 7). This study has several limitations. First, functional validation of the pathway was primarily performed using in vitro and acute mouse models; additional studies utilizing conditional gene targeting and long-term outcome assessments are warranted to further establish its translational relevance. Second, although our bioinformatic, biochemical, and functional assays strongly support the specificity of AU020206–YTHDC2 and METTL3-mediated m6A regulation, we did not perform rescue experiments using AU020206 mutants lacking YTHDC2-binding sites or catalytically inactive METTL3 mutants. Such experiments would provide more direct mechanistic validation and will be an important focus of future work. Third, although multiple independent shRNA sequences and negative control lentiviruses were used to minimize non-specific effects, potential off-target actions inherent to lentiviral knockdown and overexpression systems cannot be completely excluded. Future studies employing CRISPR-based approaches or rescue assays will be needed to further strengthen the specificity and translational potential of this pathway. Given the broader roles of m6A, lncRNAs, and ferroptosis in diverse brain pathologies, future investigations should address the potential off-target effects and the safety of manipulating this signalling network. Collectively, our data highlight a previously unrecognized METTL3-dependent epitranscriptomic safeguard in neuronal ferroptosis and provide a conceptual framework for targeting m6A–lncRNA pathways in the treatment of ischemic brain injury.

## Figures and Tables

**Table 1:** Primer sequences of genes.

Preferred Mouse Gene Name	Common Protein Name	NCBI Gene ID (Mouse)	Forward Primer (5′->3′)	Reverse Primer (5′->3′)	Amplicon (bp)
*lncRNA-AU020206*	lncRNA-AU020206	108167440	AGTGGTGATGAGGTGCTGTT	CTGAGGTAGTCTCCAGGTGC	144
*Slc7a11*	SLC7A11	26570	AGACGGTGGCAGTGTTTGTA	TGGGTTCTTCTGGGATGACA	192
*Gpx4*	GPX4	625249	TGGAGCCACGCATTTGTCAT	TCGTTCTTCAGGGACAGGAG	150
*Ythdc2*	YTHDC2	240255	CCATCTTCGACTCGCTGTTC	TGACTCGCTTGTTGTGGGTA	168
*Mettl3*	METTL3	56335	CTGAAGATGTTGGTGCCGAG	AGTGTGAGCAGGCTTTGGAT	171
*Tfrc*	TRFC	22042	TGCCTTGTGTATGCTCCACT	CAGGGAGCTGTAGGAAGGTG	180
*Acsl4*	ACSL4	50790	GATGACTTCGGGATCGTGGT	ACAGTCTGGGACCGAAAGGT	161
*Gapdh*	GAPDH	14433	AACGATTTGGTTATTG	GGAAGATGTGGTATT	130

## Data Availability

Data is contained within the article.

## Supplementary Materials

The following supporting information can be downloaded at: https://www.mdpi.com/article/10.3390/biom15101353/s1, Figure 1K, Figure 2H, Figure 3L, Figure 4A,C,D,K, Figure 5B, Figure 6C,K: Original images.

## Abbreviations

m6A	N6-methyladenosine
lncRNA	Long non-coding RNA
MCAO/R	Middle cerebral artery occlusion/reperfusion
OGD/R	Oxygen–glucose deprivation/reoxygenation
METTL3	Methyltransferase-like 3
YTHDC2	YTH domain-containing protein 2
SLC7A11	Solute carrier family 7 member 11
GPX4	Glutathione peroxidase 4
TFRC	Transferrin receptor
ACSL4	Acyl-CoA synthetase long-chain family member 4
ROS	Reactive oxygen species
PUFA	Polyunsaturated fatty acid
GSH	Glutathione
CIRI	Cerebral ischemia/reperfusion injury
qRT-PCR	Quantitative real-time polymerase chain reaction
RIP	RNA immunoprecipitation
MeRIP	Methylated RNA immunoprecipitation
SDS-PAGE	Sodium dodecyl sulphate-polyacrylamide gel electrophoresis
ELISA	Enzyme-linked immunosorbent assay
TTC	2,3,5-triphenyltetrazolium chloride
HE	Haematoxylin-eosin
TUNEL	Terminal deoxynucleotidyl transferase dUTP nick end labelling
mNSS	Modified neurological severity score

## References

[1] Feske S.K. (2021). Ischemic stroke. Am. J. Med..

[2] Mendelson S.J., Prabhakaran S. (2021). Diagnosis and management of transient ischemic attack and acute ischemic stroke: A review. JAMA.

[3] Qureshi A.I., Baskett W.I., Huang W., Shyu D., Myers D., Raju M., Lobanova I., Suri M.F.K., Naqvi S.H., French B.R. (2021). Acute ischemic stroke and COVID-19: An analysis of 27 676 patients. Stroke.

[4] Sarraj A., Hassan A.E., Abraham M.G., Ortega-Gutierrez S., Kasner S.E., Hussain M.S., Chen M., Blackburn S., Sitton C.W., Churilov L. (2023). Trial of endovascular thrombectomy for large ischemic strokes. N. Engl. J. Med..

[5] Rabinstein A.A. (2020). Update on treatment of acute ischemic stroke. Contin. Lifelong Learn. Neurol..

[6] Jurcau A., Simion A. (2021). Neuroinflammation in cerebral ischemia and ischemia/reperfusion injuries: From pathophysiology to therapeutic strategies. Int. J. Mol. Sci..

[7] Sendinc E., Shi Y. (2023). RNA m6A methylation across the transcriptome. Mol. Cell.

[8] Jiang L., Lin W., Zhang C., Ash P.E.A., Verma M., Kwan J., van Vliet E., Yang Z., Cruz A.L., Boudeau S. (2021). Interaction of tau with HNRNPA2B1 and N(6)-methyladenosine RNA mediates the progression of tauopathy. Mol. Cell.

[9] Xu K., Mo Y., Li D., Yu Q., Wang L., Lin F., Kong C., Balelang M.F., Zhang A., Chen S. (2020). N(6)-methyladenosine demethylases Alkbh5/Fto regulate cerebral ischemia-reperfusion injury. Ther. Adv. Chronic Dis..

[10] Sheng Y., Wei J., Yu F., Xu H., Yu C., Wu Q., Liu Y., Li L., Cui X.L., Gu X. (2021). A critical role of nuclear m6A reader YTHDC1 in leukemogenesis by regulating MCM complex-mediated DNA replication. Blood.

[11] Wu X., Liu H., Wang J., Zhang S., Hu Q., Wang T., Cui W., Shi Y., Bai H., Zhou J. (2025). The m(6)A methyltransferase METTL3 drives neuroinflammation and neurotoxicity through stabilizing BATF mRNA in microglia. Cell Death Differ..

[12] Bridges M.C., Daulagala A.C., Kourtidis A. (2021). LNCcation: lncRNA localization and function. J. Cell Biol..

[13] Xu S., Li Y., Chen J.P., Li D.Z., Jiang Q., Wu T., Zhou X.Z. (2020). Oxygen glucose deprivation/re-oxygenation-induced neuronal cell death is associated with Lnc-D63785 m6A methylation and miR-422a accumulation. Cell Death Dis..

[14] Chen L., Sun K., Qin W., Huang B., Wu C., Chen J., Lai Q., Wang X., Zhou R., Li A. (2023). LIMK1 m(6)A-RNA methylation recognized by YTHDC2 induces 5-FU chemoresistance in colorectal cancer via endoplasmic reticulum stress and stress granule formation. Cancer Lett..

[15] Saito Y., Hawley B.R., Puno M.R., Sarathy S.N., Lima C.D., Jaffrey S.R., Darnell R.B., Keeney S., Jain D. (2022). YTHDC2 control of gametogenesis requires helicase activity but not m(6)A binding. Genes Dev..

[16] Jiang X., Stockwell B.R., Conrad M. (2021). Ferroptosis: Mechanisms, biology and role in disease. Nat. Rev. Mol. Cell Biol..

[17] Liang D., Minikes A.M., Jiang X. (2022). Ferroptosis at the intersection of lipid metabolism and cellular signaling. Mol. Cell.

[18] Yuan Y., Zhai Y., Chen J., Xu X., Wang H. (2021). Kaempferol Ameliorates Oxygen-Glucose Deprivation/Reoxygenation-Induced Neuronal Ferroptosis by Activating Nrf2/SLC7A11/GPX4 Axis. Biomolecules.

[19] Wang L., Liu Y., Du T., Yang H., Lei L., Guo M., Ding H.F., Zhang J., Wang H., Chen X. (2020). ATF3 promotes erastin-induced ferroptosis by suppressing system Xc^−^. Cell Death Differ..

[20] Yin F., Liu K., Peng W., Jiang D., Zhang H., Guo P., Wu Y., Zhang X., Sun C., Wang Y. (2023). The Effect of N6-Methyladenosine Regulators and m6A Reader YTHDC1-Mediated N6-Methyladenosine Modification Is Involved in Oxidative Stress in Human Aortic Dissection. Oxidative Med. Cell. Longev..

[21] Zhang H., Zan J., Zhong K., Lu M., Sun X., Tan W. (2018). Neuroprotective Effects of Isosteviol Sodium through Increasing CYLD by the Downregulation miRNA-181b. Brain Res. Bull..

[22] Zhang H., Sun X., Xie Y., Zan J., Tan W. (2017). Isosteviol Sodium Protects Against Permanent Cerebral Ischemia Injury in Mice via Inhibition of NF-κB–Mediated Inflammatory and Apoptotic Responses. J. Stroke Cerebrovasc. Dis..

[23] Zhang H., Sun X., Xie Y., Tian F., Hu H., Tan W. (2018). Isosteviol sodium inhibits astrogliosis after cerebral ischemia/reperfusion injury in rats. Biol. Pharm. Bull..

[24] Zeng L.L., He X.S., Liu J.R., Zheng C.B., Wang Y.T., Yang G.Y. (2016). Lentivirus-Mediated Overexpression of MicroRNA-210 Improves Long-Term Outcomes after Focal Cerebral Ischemia in Mice. CNS Neurosci. Ther..

[25] Chen J., Li Y., Wang L., Zhang Z., Lu D., Lu M., Chopp M. (2001). Therapeutic benefit of intravenous administration of bone marrow stromal cells after cerebral ischemia in rats. Stroke.

[26] Hui H., Xiao O.S., Fang T., Hao Z., Liu Q., Wen T. (2016). Neuroprotective Effects of Isosteviol Sodium Injection on Acute Focal Cerebral Ischemia in Rats. Oxidative Med. Cell. Longev..

[27] Guo Y., Yang J.H., He Y., Zhou H.F., Wang Y., Ding Z.S., Jin B., Wan H.T. (2022). Protocatechuic aldehyde prevents ischemic injury by attenuating brain microvascular endothelial cell pyroptosis via lncRNA Xist. Phytomedicine.

[28] Gupta G., Bhat A.A., Goyal A., Singla N., Gupta S., Sharma S., Bhatt S., Dua K. (2023). Exploring ACSL4/LPCAT3/ALOX15 and SLC7A11/GPX4/NFE2L2 as potential targets in ferroptosis-based cancer therapy. Futur. Med. Chem..

[29] Xie C.J., Gu A.P., Cai J., Wu Y., Chen R.C. (2018). Curcumin protects neural cells against ischemic injury in N2a cells and mouse brain with ischemic stroke. Brain Behav..

[30] Chen Z., Hu W., Wang J., Rao D., Zhu J. (2024). Knockdown of lncRNA AU020206 could inhibit microglia apoptosis in ischemic stroke. Cell. Mol. Biol..

[31] Bai Y., Dai X., Ye T., Zhang P., Yan X., Gong X., Liang S., Chen M. (2019). PlncRNADB: A repository of plant lncRNAs and lncRNA-RBP protein interactions. Curr. Bioinform..

[32] Wu X., Chen H., Li K., Zhang H., Li K., Tan H. (2024). The biological function of the N6-Methyladenosine reader YTHDC2 and its role in diseases. J. Transl. Med..

[33] Puvvula P.K. (2019). LncRNAs Regulatory Networks in Cellular Senescence. Int. J. Mol. Sci..

[34] Baruah C., Nath P., Barah P. (2022). LncRNAs in neuropsychiatric disorders and computational insights for their prediction. Mol. Biol. Rep..

[35] Srinivas T., Mathias C., Oliveira-Mateos C., Guil S. (2023). Roles of lncRNAs in brain development and pathogenesis: Emerging therapeutic opportunities. Mol. Ther..

[36] Wang L., Li S., Stone S.S., Liu N., Gong K., Ren C., Sun K., Zhang C., Shao G. (2022). The Role of the lncRNA MALAT1 in Neuroprotection against Hypoxic/Ischemic Injury. Biomolecules.

[37] Cao Y., Liu J., Lu Q., Huang K., Yang B., Reilly J., Jiang N., Shu X., Shang L. (2022). An update on the functional roles of long non-coding RNAs in ischemic injury (Review). Int. J. Mol. Med..

[38] Yang J., Chen M., Cao R.Y., Li Q., Zhu F. (2018). The Role of Circular RNAs in Cerebral Ischemic Diseases: Ischemic Stroke and Cerebral Ischemia/Reperfusion Injury. Adv. Exp. Med. Biol..

[39] Zhang C., Zhang X., Gong Y., Li T., Yang L., Xu W., Dong L. (2020). Role of the lncRNA-mRNA network in atherosclerosis using ox-low-density lipoprotein-induced macrophage-derived foam cells. Mol. Omics.

[40] Chen C., Huang Y., Xia P., Zhang F., Li L., Wang E., Guo Q., Ye Z. (2021). Long noncoding RNA Meg3 mediates ferroptosis induced by oxygen and glucose deprivation combined with hyperglycemia in rat brain microvascular endothelial cells, through modulating the p53/GPX4 axis. Eur. J. Histochem..

[41] Lu J., Xu F., Lu H. (2020). LncRNA PVT1 regulates ferroptosis through miR-214-mediated TFR1 and p53. Life Sci..

[42] Dykes I.M., Emanueli C. (2017). Transcriptional and post-transcriptional gene regulation by long non-coding RNA. Genom. Proteom. Bioinform..

[43] Wang H., Yuan J., Dang X., Shi Z., Ban W., Ma D. (2021). Mettl14-mediated m6A modification modulates neuron apoptosis during the repair of spinal cord injury by regulating the transformation from pri-mir-375 to miR-375. Cell Biosci..

[44] Song J., Hao J., Lu Y., Ding X., Li M., Xin Y. (2024). Increased m(6)A modification of BDNF mRNA via FTO promotes neuronal apoptosis following aluminum-induced oxidative stress. Environ. Pollut..

[45] Liu S., Zhuo L., Wang J., Zhang Q., Li Q., Li G., Yan L., Jin T., Pan T., Sui X. (2020). METTL3 plays multiple functions in biological processes. Am. J. Cancer Res..

[46] Anders M., Chelysheva I., Goebel I., Trenkner T., Zhou J., Mao Y., Verzini S., Qian S.B., Ignatova Z. (2018). Dynamic m(6)A methylation facilitates mRNA triaging to stress granules. Life Sci. Alliance.

[47] Hsu P.J., Zhu Y., Ma H., Guo Y., Shi X., Liu Y., Qi M., Lu Z., Shi H., Wang J. (2017). Ythdc2 is an N6-methyladenosine binding protein that regulates mammalian spermatogenesis. Cell Res..

[48] Tang J., Tang Q.-X., Liu S. (2023). METTL3-modified lncRNA-SNHG8 binds to PTBP1 to regulate ALAS2 expression to increase oxidative stress and promote myocardial infarction. Mol. Cell. Biochem..

[49] He F., Zhang P., Liu J., Wang R., Kaufman R.J., Yaden B.C., Karin M. (2023). ATF4 suppresses hepatocarcinogenesis by inducing SLC7A11 (xCT) to block stress-related ferroptosis. J. Hepatol..

[50] Fu C., Wu Y., Liu S., Luo C., Lu Y., Liu M., Wang L., Zhang Y., Liu X. (2022). Rehmannioside A improves cognitive impairment and alleviates ferroptosis via activating PI3K/AKT/Nrf2 and SLC7A11/GPX4 signaling pathway after ischemia. J. Ethnopharmacol..

[51] Liu H., Zhang T.A., Zhang W.Y., Huang S.R., Hu Y., Sun J. (2023). Rhein attenuates cerebral ischemia-reperfusion injury via inhibition of ferroptosis through NRF2/SLC7A11/GPX4 pathway. Exp. Neurol..

[52] Liu H., Yue Q., Zhang W., Ding Q., Yang J., Lin M., Sun J. (2024). Xinglou Chengqi Decoction Protects against Cerebral Ischemia/Reperfusion Injury by Inhibiting Ferroptosis via SLC7A11/GPX4 Signaling. Adv. Biol..

[53] Liu T., Cui Y., Dong S., Kong X., Xu X., Wang Y., Wan Q., Wang Q. (2022). Treadmill Training Reduces Cerebral Ischemia-Reperfusion Injury by Inhibiting Ferroptosis through Activation of SLC7A11/GPX4. Oxidative Med. Cell. Longev..

