# Supplementary figures and images for: METTL3-Driven m6A Epigenetic Remodeling of lncRNA-AU020206 Stabilizes SLC7A11 via YTHDC2 Attenuates Apoptosis and Ferroptosis in Cerebral Ischemia/Reperfusion Injury
Source: Biomolecules. 2025 Sep 24;15(10):1353. doi: 10.3390/biom15101353 (PMC12564424; doi:10.3390/biom15101353)

Fig.1K

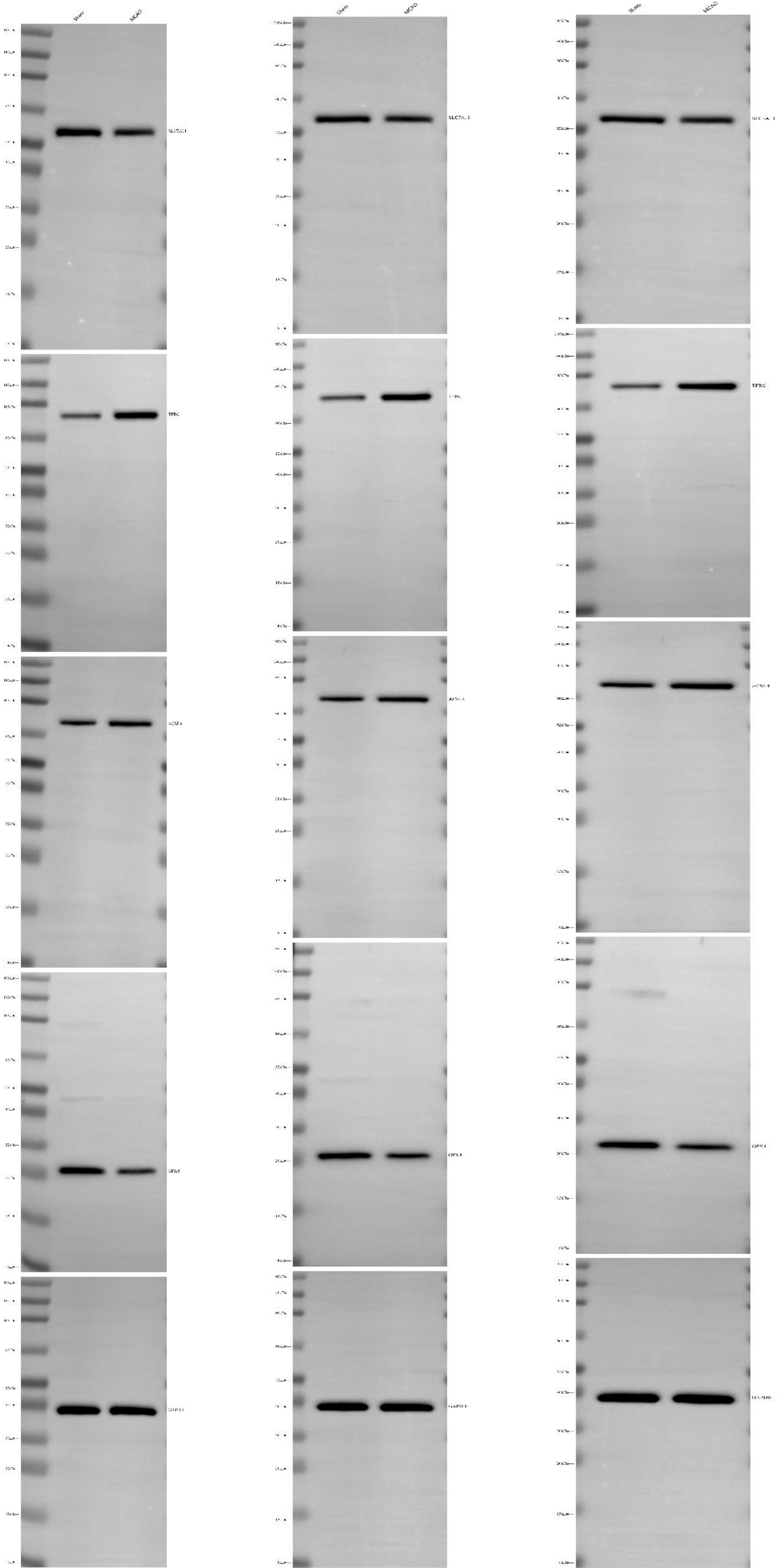

Fig.2H

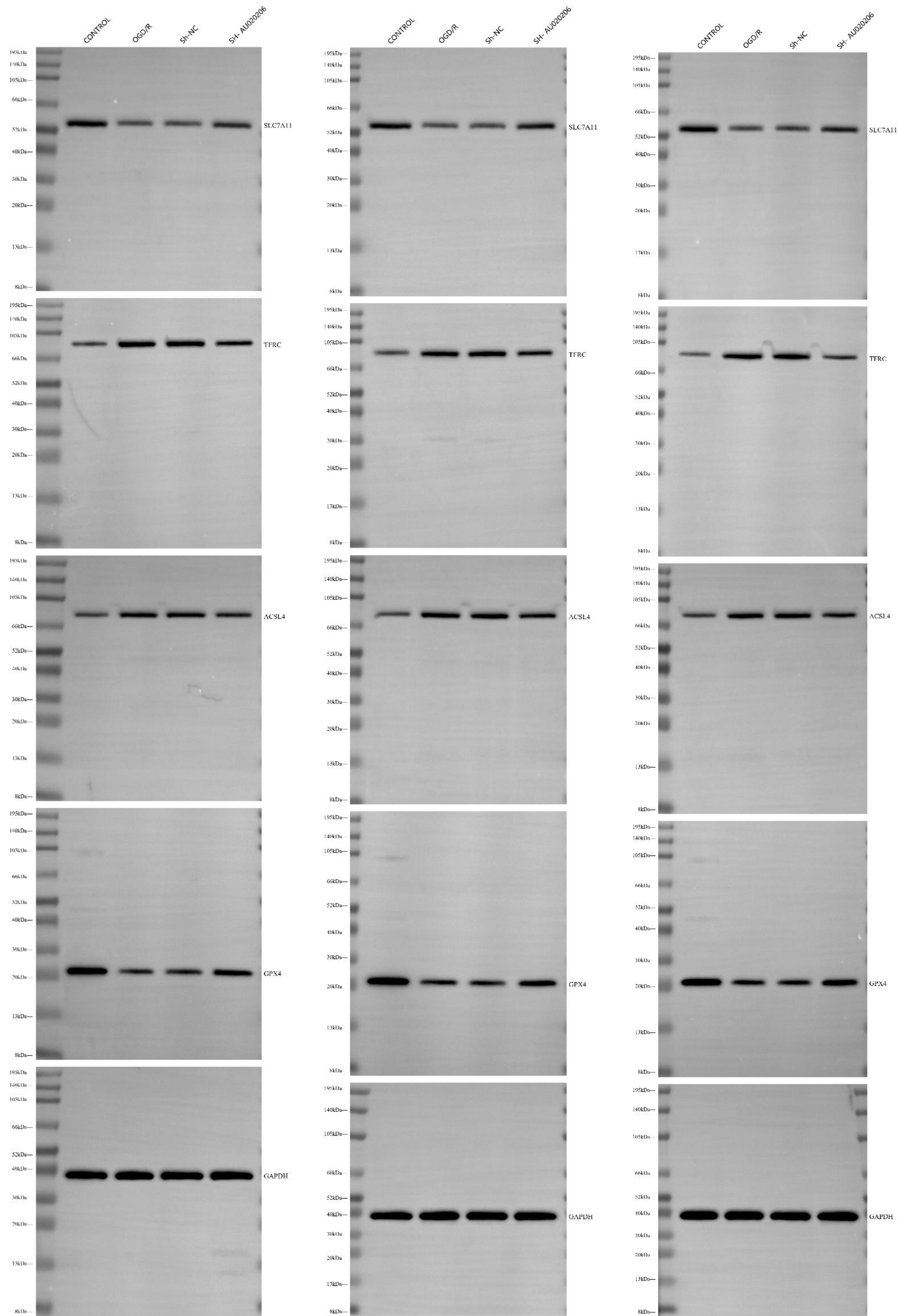

Fig.3L

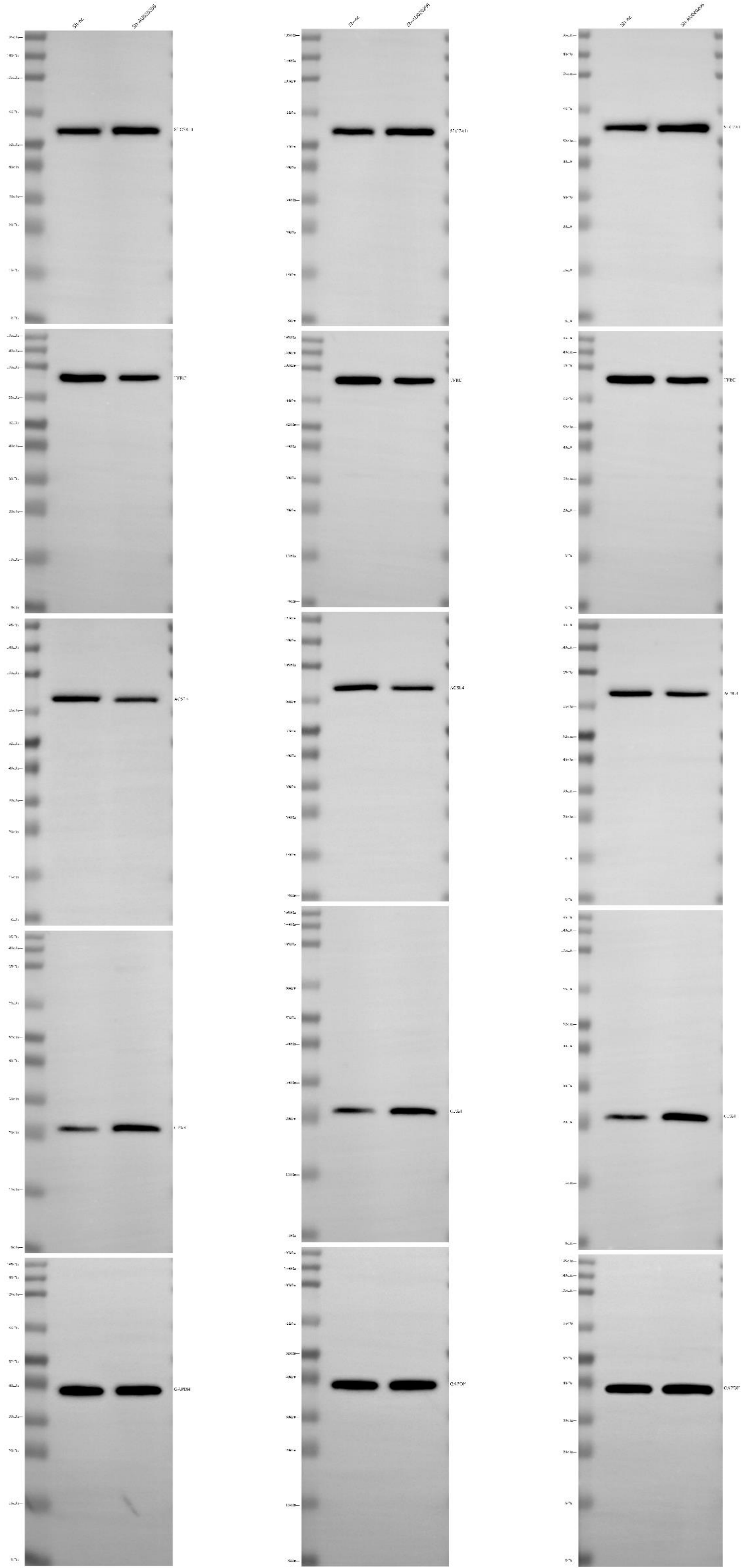

Fig.4A, 4C and 4D

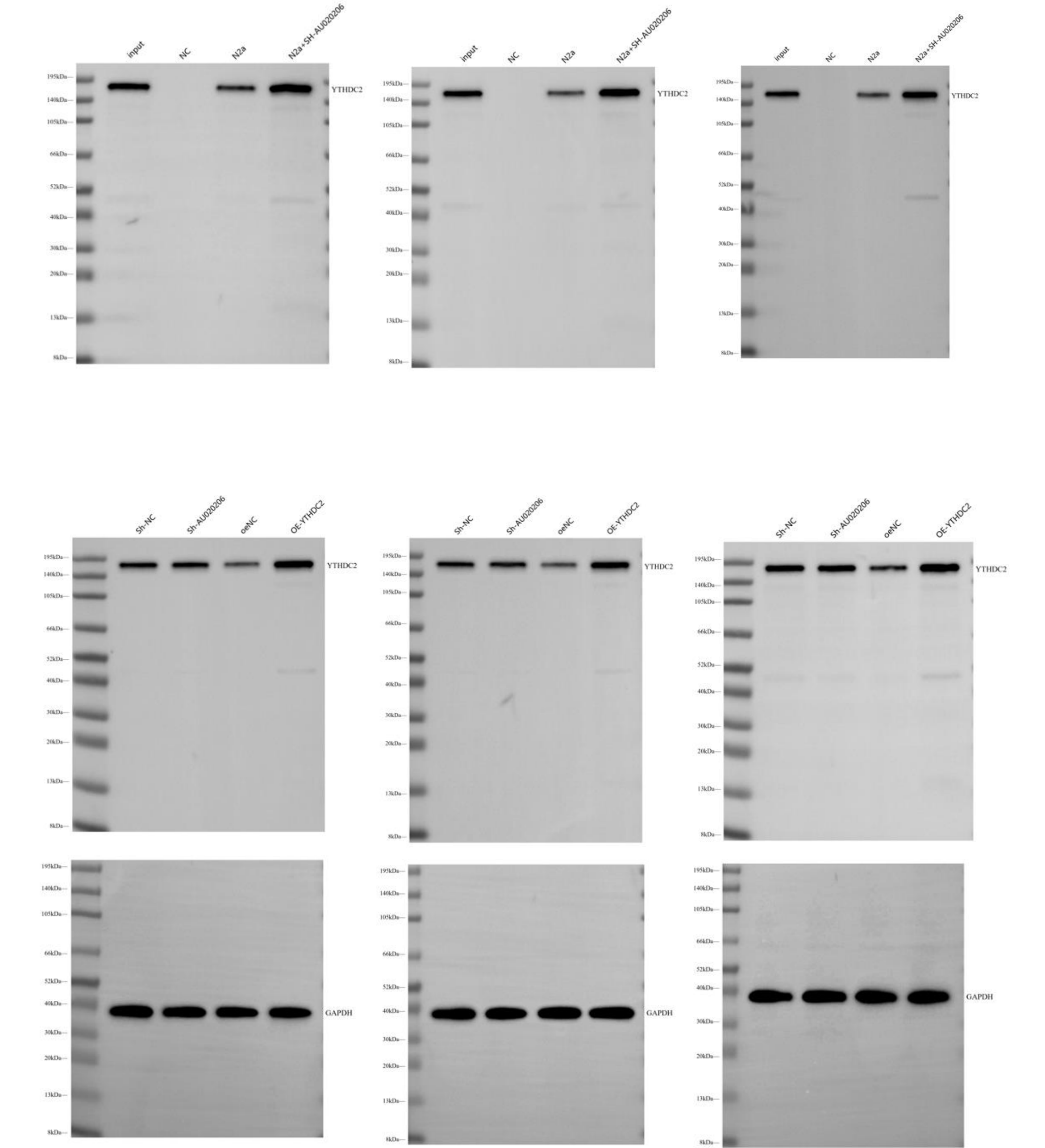

Fig.4K and Fig.5B

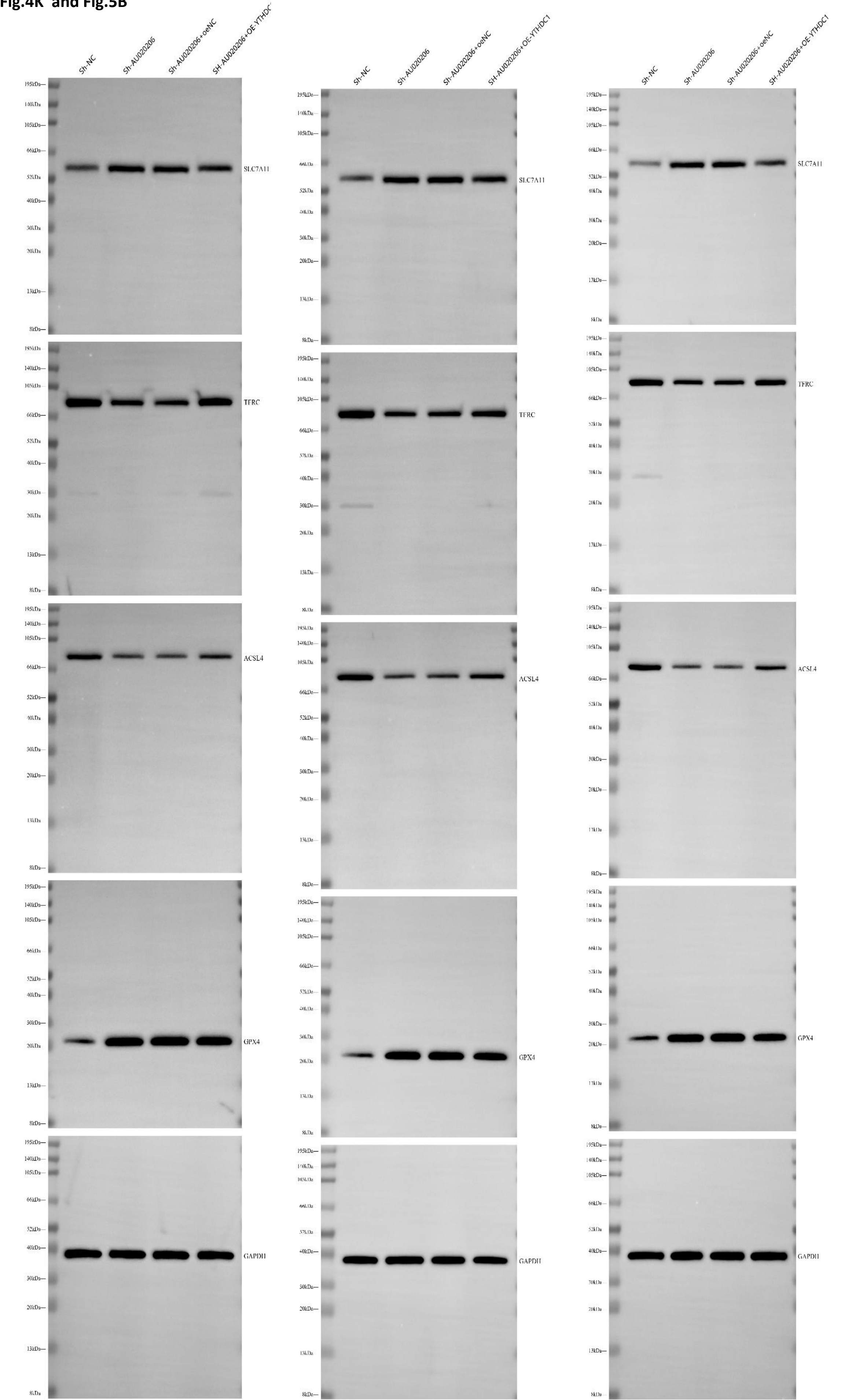

Fig.6C

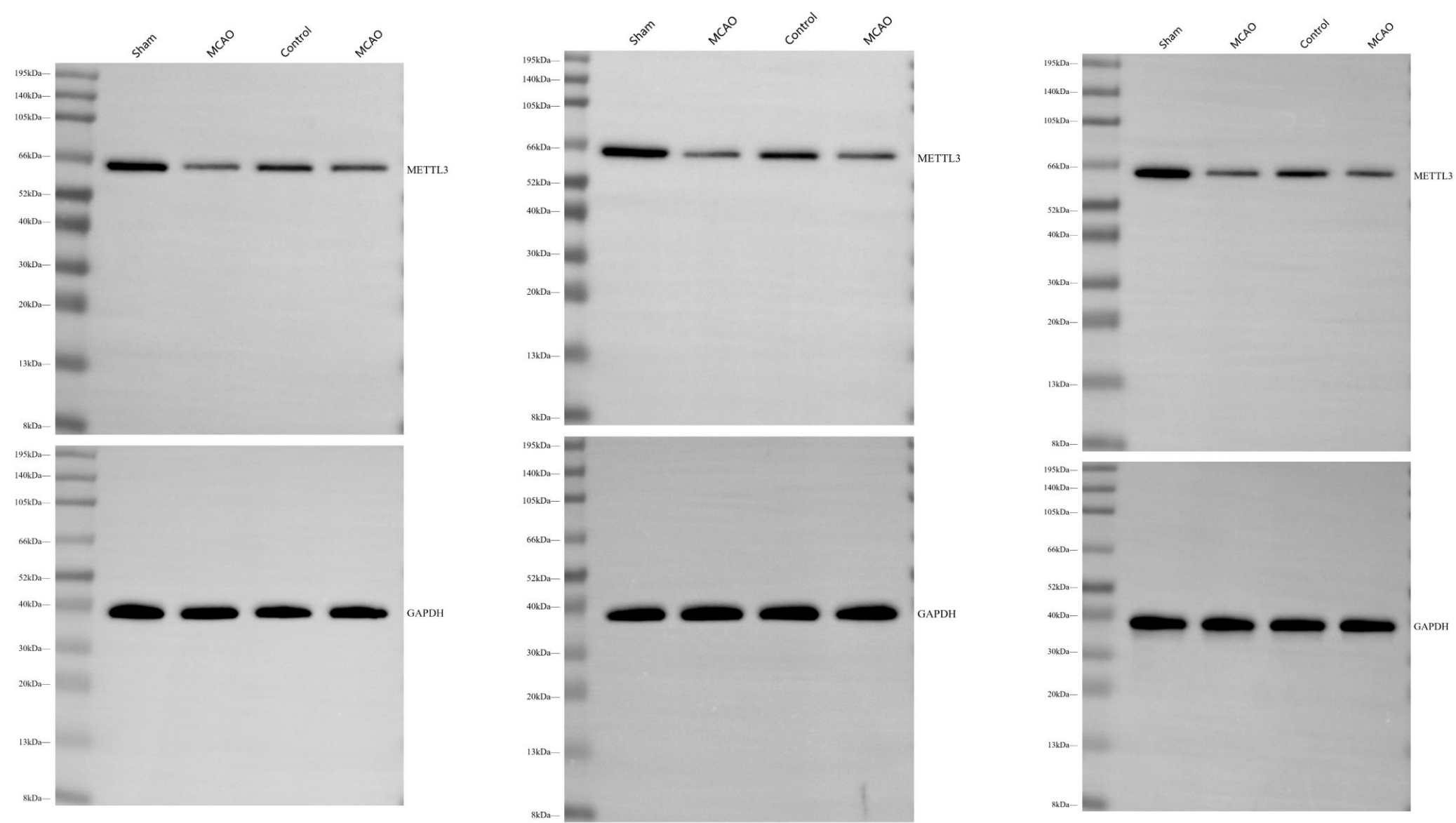

Fig.6K

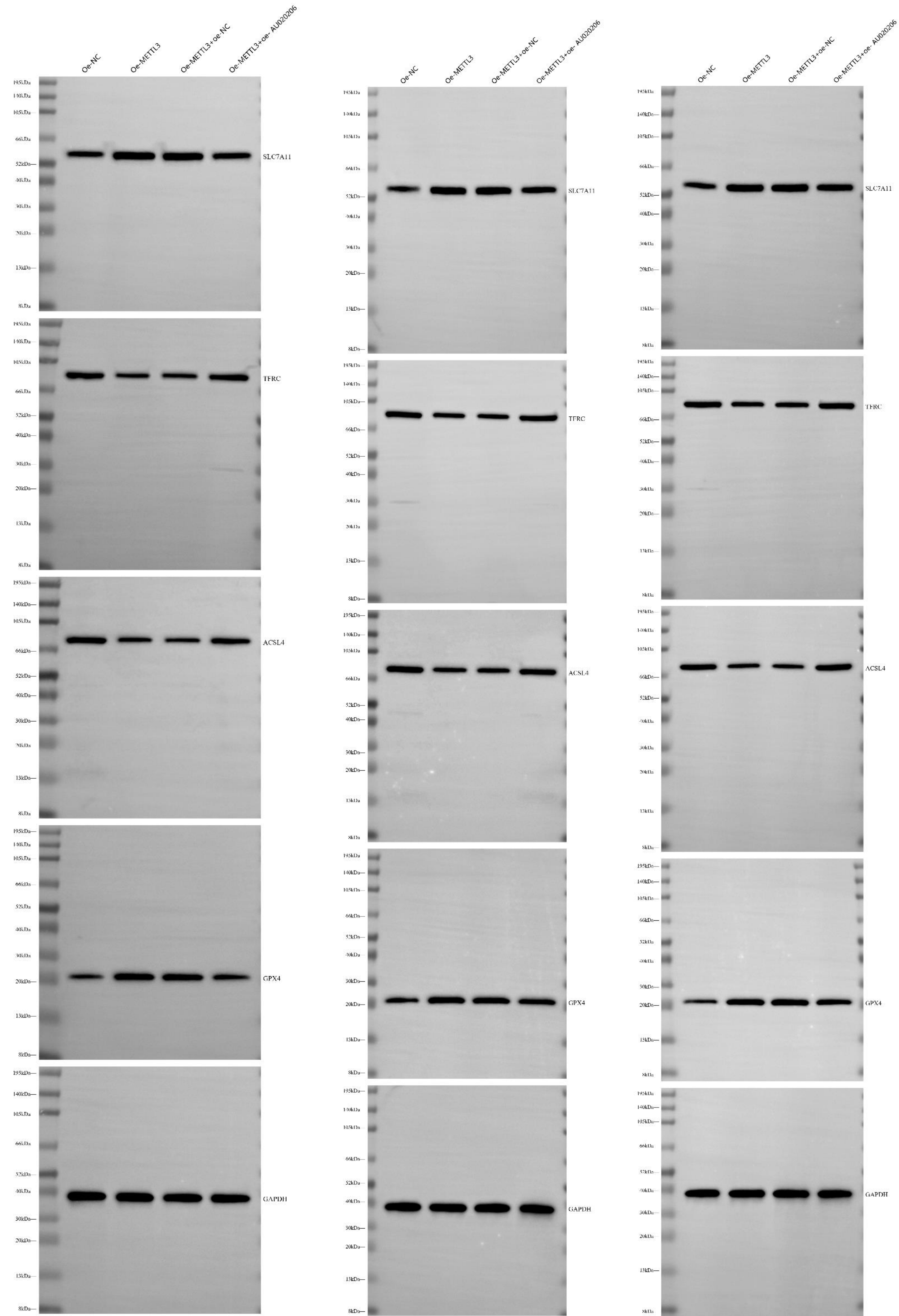

Supplement: Supplementary file 1 [file biomolecules-15-01353-s001.zip › biomolecules-3873062-supplementary.pdf]
